# Leber's Hereditary Optic Neuropathy with Mitochondrial DNA Mutation G11778A: A Systematic Literature Review and Meta-Analysis

**DOI:** 10.1155/2023/1107866

**Published:** 2023-01-24

**Authors:** Jiajia Yuan, Jiaxun Zhao, Chong Ye, Long Pang, Xin Zhang, Alvin Luk, Yangyang Du, Kai Yoon Fan, Xiaowen Zhang, Bin Li, Changzheng Chen

**Affiliations:** ^1^Department of Ophthalmology, Renmin Hospital of Wuhan University, Wuhan, China; ^2^Real-World Solutions, IQVIA, Shanghai, China; ^3^Wuhan Neurophth Biotechnology Co., Ltd., Wuhan, China; ^4^Neurophth Therapeutics, Inc., Boston, USA; ^5^Tongji Hospital, Tongji Medical College of HUST, Wuhan, China

## Abstract

**Background:**

LHON is a progressive disease with early disease onset and male predominance, usually causing devastating visual loss to patients. These systematic review and meta-analysis are aimed at summarizing epidemiology, disease onset and progression, visual recovery, risk factors, and treatment options of Leber's hereditary optic neuropathy (LHON) with mitochondrial DNA mutation G11778A from current evidence.

**Methods:**

The PubMed database was examined from its inception date to November 2021. Data from included studies were pooled with either a fixed-effects model or a random-effects model, depending on the results of heterogeneity tests. Sensitivity analysis was conducted to test the robustness of results.

**Results:**

A total of 41 articles were included in the systematic review for qualitative analysis, and 34 articles were included for quantitative meta-analysis. The pooled estimate of proportion of G11778A mutation among the three primary mutations of mitochondrial DNA (G11778A, G3460A, and T14484C) for LHON was 73% (95% CI: 67% and 79%), and the LHON patients with G11778A mutation included the pooled male ratio estimate of 77% (76% and 79%), the pooled age estimate of 35.3 years (33.2 years and 37.3 years), the pooled onset age estimate of 22.1 years (19.7 years and 24.6 years), the pooled visual acuity estimate of 1.4 LogMAR (1.2 LogMAR and 1.6 LogMAR), and the pooled estimate of spontaneous visual recovery rate (in either 1 eye) of 20% (15% and 27%).

**Conclusions:**

The G11778A mutation is a prevalent mitochondrial DNA mutation accounting for over half of LHON cases with three primary mutations. Spontaneous visual recovery is rare, and no effective treatment is currently available.

## 1. Introduction

Leber's hereditary optic neuropathy (LHON) is one of the most common inherited mitochondrial disorders caused by mutation in the mitochondrial DNA (mtDNA) with progressive central vision loss. The typical age of disease onset is between 15 and 35 years [1]. Males are more likely to be affected by LHON than females, with the ratio reported to range from 4 : 1 to 5 : 1 [2].

Three primary pathogenic point mtDNA mutations for LHON are G11778A, G3460A, and T14484C correlated in the NADH dehydrogenase subunit 4 (*ND4*), *ND1*, and *ND6* genes of complex I in the electron transport chain, respectively. These three mutations are responsible for over 90% of LHON cases [3, 4], among which the G11778A mutation is the most common, followed by the G3460A and T14484C. The prevalence of LHON in northern Europeans ranged from 1/50,000 to 1/30,000 [2, 5, 6], and around 57% of LHON patients have G11778A mutation [7]. In China, a study reported the percentage of LHON patients with G11778A mutation as high as 90% [8].

Overall, the visual prognosis of LHON is poor, and the majority of patients will remain visually impaired for the rest of their lives [9]. Among the three primary mtDNA mutations for LHON, G11778A has been reported to be associated with worse visual prognosis compared to the other two mutations [10]. Besides clinical burden, LHON has severe negative impact on patients' quality of life and individuals with G11778A mutation have significantly worse quality of life compared to those with T14484C mutation [11]. In addition, some studies have reported that LHON can be associated with cardiac and neurologic disorders [12–14]. Currently, there is no proven effective treatment for LHON even though several therapies have been tested [15]. The effectiveness of existing treatments still needs to be further explored [16].

Although LHON is a complex disease which involves interactions between genetic modifications and environmental factors, information for LHON patients with G11778A mutation has not been comprehensively summarized and discussed. Here, we conducted these systematic review and meta-analysis to summarize the current research and existing evidence to understand the epidemiology, disease onset and progression, visual recovery, risk factors, and treatment options of G11778A LHON.

## 2. Methods

We undertook this systematic review according to the guidelines of Preferred Reporting Items for Systematic Reviews and Meta-analyses (PRISMA) [17]. This review adhered to the Declaration of Helsinki. Since this was a systematic review article with meta-analyses of published studies without involving human subjects, no informed consent from patients was needed.

### 2.1. Source and Search Methods

We designed a comprehensive search strategy to retrieve relevant data from published literatures, and the search strategy could be found in the review protocol (Supplementary 1). We searched Medline (PubMed) database for scientific literatures written in English and published from its inception date to November 2021. A combination of search terms was applied, including keywords related to LHON epidemiology, diagnosis/testing, treatment, prognosis, quality of life, and clinical guideline/consensus. The search language was limited to English. All search result citations were imported into and managed by the EndNote bibliographic software.

### 2.2. Study Selection

Published epidemiological studies with population of interest including LHON patients with G11778A mutation would be included. The study selection process was independently performed by two reviewers based on specific questions (Supplementary 2). Any disagreements between the two reviewers were resolved by discussion to reach consensus. If consensus could not be reached, a third reviewer would independently appraise the article and discuss it with the two reviewers to reach consensus.

### 2.3. Data Collection and Study Quality

Data extraction was performed by the topic leader and corroborated by a secondary reviewer for quality assurance. Data on study information (title, year of publication, journal, volume, issue, page, digital object identifier, publication type, study design, sample size, and study location), participants' characteristics (population definition, age, gender, and ethnicity), disease epidemiology (incidence rate, prevalence rate, and mutation rate), disease characteristics (family history, gender ratio/bias, onset age, disease natural history, visual acuity (VA), contrast sensitivity, and visual field), diagnosis and treatment (diagnosis/testing, treatment/intervention, and adverse effects of treatment/intervention), quality of life, and health economics were extracted, if available. The risk of bias and quality of studies were assessed by two reviewers using Joanna Briggs Institute model of evidence-based healthcare bias assessment [18]. Disputes between reviewers were resolved through discussion until consensus was reached.

### 2.4. Data Synthesis and Statistical Analysis

Outcomes reported by included studies were recorded in standardized data extraction forms. Results of the included studies were pooled where appropriate with either a fixed-effects model for low heterogeneity or a random-effects model (Sidik-Jonkman's estimator) [19] for moderate or high heterogeneity to calculate the summarized effects and 95% confidence interval (CI). Heterogeneity across studies for each selected outcome was evaluated using the Cochran *Q* statistic and quantified with *I*^2^ statistic. Significant heterogeneity was considered when either *P* < 0.05 in Cochran *Q* test or *I*^2^ ≥ 50%. The classification of heterogeneity was based on *I*^2^ statistic, with *I*^2^ of 25% indicating low level of heterogeneity, 50% indicating moderate level of heterogeneity, and 75% indicating high level of heterogeneity [20]. Sensitivity analysis was conducted to test the robustness of the meta-analysis. A leave-one-out analysis was performed first by excluding one study at a time and reperforming meta-analysis to evaluate if the pooled result falls outside of the 95% CI of the overall summary measurement. Three algorithms (i.e., *K*-means clustering, density-based spatial clustering of applications with noise (DBSCAN), and Gaussian mixture models) were applied to identify the potential outliers. The Baujat plot was used to assist in potential outlier identification by visualizing the studies located at the right side of the plot, meaning considerable contribution to either the heterogeneity or pooled summary measurement [21]. The meta-analysis was reperformed after the exclusion of potential outliers. Further descriptive analysis on available individual data from included studies would be conducted to summarize clinical characteristics if available. Statistical software R (version 3.6.1) was used to perform statistical analysis [22]. A *P* value of less than 0.05 was considered statistically significant difference.

### 2.5. Patient and Public Involvement

Patients or the public was not involved in the design, or conduct, or reporting, or dissemination plans of our research.

## 3. Results

### 3.1. Study Selection

**Figure 1:**
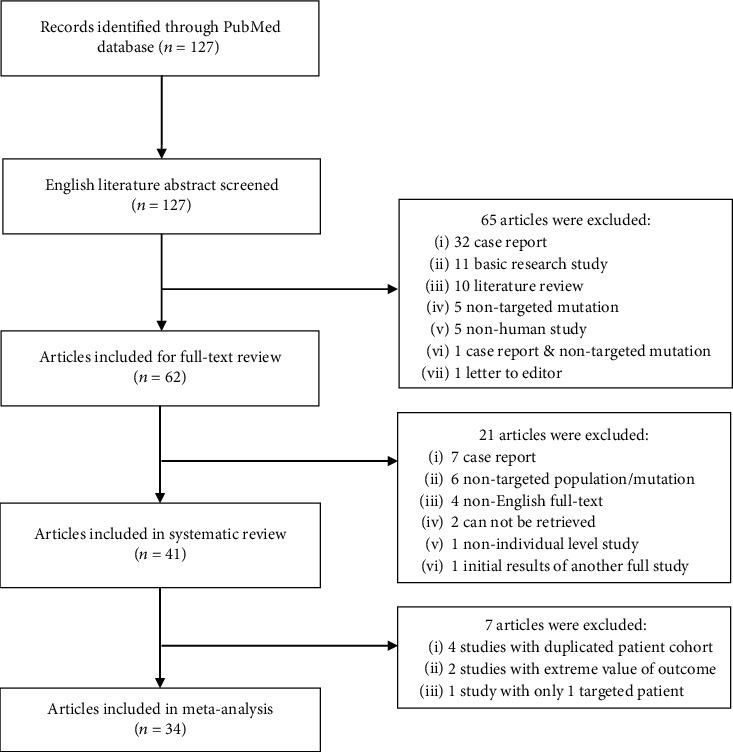
Flow chart of article selection.

Initial search identified a total of 127 articles without duplicates. Based on screening of the titles and abstracts, 62 articles were considered potentially eligible and were included for full-text review, after which 7 case reports [23–29], 6 articles without targeted population or mutation [30–35], 2 articles without full-text availability [36, 37], 4 articles without English full-text availability [38–41], 1 article with nonindividual level study [42], and 1 article reporting initial results [43] of another included article [44] were finally excluded. At last, a total of 41 articles were included in the systematic review for qualitative analysis. General characteristics of the included studies are presented in Table S1. For further meta-analysis, 4 articles [45–48] investigating the same patient cohort as other included articles [49, 50], 1 article [51] with only 1 G11778A mutation carrier converting from carrier to affected patient, and 2 articles [52, 53] reporting extreme value of proportion of G11778A among the three primary mutations were excluded. A total of 34 articles were finally included in quantitative meta-analysis.  Figure 1 shows the detailed flow of articles included at each step during the review process.

### 3.2. Study Quality

We identified 31 case series studies, among which 26 studies were included in our meta-analysis. Two studies reporting LHON prevalence data and 8 trials were subsequently identified and included. Overall, patient's inclusion criteria were not clearly described in most included case series studies, and the status of complete inclusion remained unclear. Trials and studies reporting prevalence were in relatively good qualities. A summary of bias is provided in Supplementary 3.

### 3.3. Proportion of G11778A Mutation among the Three Primary LHON Mutations

**Figure 2:**
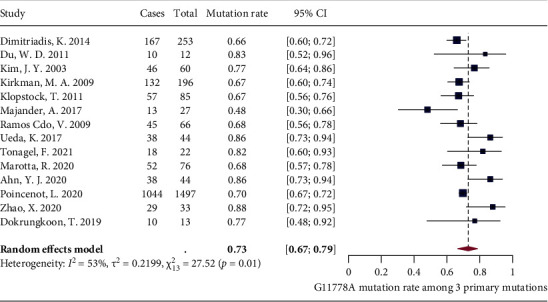
Forest plot of G11778A mutation rate.

In addition to studies that only included LHON patients with G11778A mutation, there were a number of studies including LHON patients with the other two primary mutations (G3460A and T14484C). A total of 20 studies reported the proportion of G11778A mutation among LHON patients harboring one of the three primary mtDNA mutations. Among these studies, three have not been included in the meta-analysis by showing the extreme dominance of G11778A among the three primary mutations with the reported G11778A proportion of 100% (11 out of 11), 97.4% (38 out of 39), and 95.8% (23 out of 24) [52–54]. Meta-analysis was first performed on the remaining 17 studies, and significant heterogeneity was detected (*I*^2^ = 88%, *P* < 0.01) (Supplementary 4-A). Leave-one-out analysis was conducted subsequently as sensitivity analysis, and it revealed that all the pooled estimates after omitting one study at a time were within the 95% CI of the overall estimate (Supplementary 4-B). After excluding two outliers [55, 56] identified by the three algorithms (i.e., *K*-means clustering, DBSCAN, and Gaussian mixture models) (Supplementary 4-C), the meta-analysis was reperformed on the remaining 15 studies and the heterogeneity remained significant (*I*^2^ = 65%, *P* < 0.01). We further explored the influence of each study by plotting the Baujat plot (Supplementary 4-D), and 1 study [57] still lay at the right side of the plot. Exclusion of this potential outlier led to decline in heterogeneity (*I*^2^ = 53%, *P* = 0.01), and random-effects model showed that the pooled proportion of G11778A mutation was 73% (67% and 79%) (Figure 2).

Among the 14 studies, country-level data of G11778A mutation proportion among the three primary mutations was available for 13 studies from which we merged the mutation proportion data by different geographical regions including Europe, the Asia-Pacific, the Middle East, South America, North America, and Africa. Based on the aggregated results of the region subgroups, the proportion of G11778A mutation was 75% (346/460 patients) for the Asia-Pacific, 75% (3/4 patients) for Africa, 68% (660/963 patients) for Europe, 68% (71/105 patients) for South America, 68% (557/818 patients) for North America, and 57% (4/7 patients) for the Middle East.

**Figure 3:**
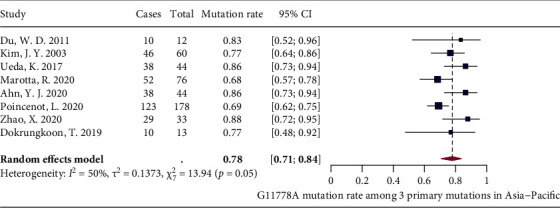
Forest plot of G11778A mutation rate in Asia-Pacific.

**Figure 4:**
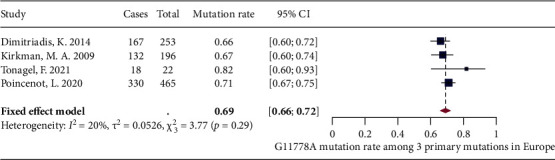
Forest plot of G11778A mutation rate in Europe.

Due to limited number of studies covering Africa, South America, North America, and the Middle East, meta-analyses of G11778A mutation proportion were only performed on studies conducted in Asia-Pacific (8 studies) and Europe (5 studies). For Asia-Pacific region, random-effects model showed that the pooled proportion of G11778A mutation was 78% (71% and 84%) with moderate heterogeneity (*I*^2^ = 50%, *P* = 0.05) (Figure 3). For Europe, after excluding one outlier identified by the three algorithms, fixed-effects model showed that the pooled proportion of G11778A mutation was 69% (66% and 72%) with low heterogeneity (*I*^2^ = 20%, *P* = 0.29) (Figure 4).

### 3.4. Male Ratio of G11778A LHON Patients

**Figure 5:**
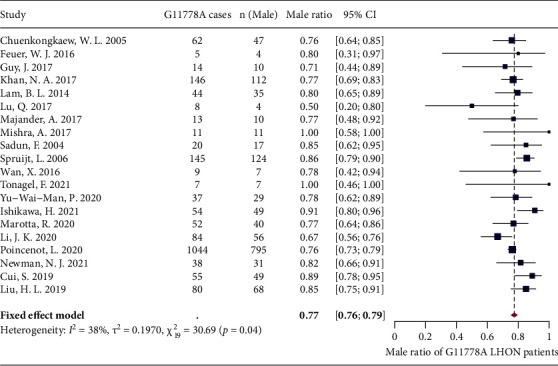
Forest plot of male ratio among of G11778A LHON patients.

A total of 24 studies reported the male ratio among their G11778A LHON patient cohort. Meta-analysis was first performed on included studies with significant heterogeneity detected (*I*^2^ = 65%, *P* < 0.01) (Supplementary 5-A). Leave-one-out analysis showed that all the pooled estimates after omitting one study at a time were within the 95% CI of the overall estimate (Supplementary 5-B). By applying the three algorithms, 4 potential outliers [58–61] were identified (Supplementary 5-C), and the heterogeneity dropped significantly for the meta-analysis reperformed after excluding these outliers (*I*^2^ = 38%, *P* = 0.10). The fixed-effects model showed that the pooled estimate of male ratio for G11778A LHON patients was 77% (76% and 79%) (Figure 5).

### 3.5. Age of G11778A LHON Patients

**Figure 6:**
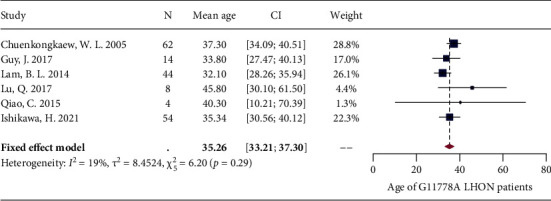
Forest plot of patient age.

A total of 12 studies reported the patient age at enrolment. Among these studies, one study was not included in the meta-analysis since it only reported the mean of age without reporting the standard deviation (SD) [62]. Meta-analysis was first performed on the remaining 11 studies showing significant heterogeneity (*I*^2^ = 87%, *P* < 0.01) (Supplementary 6-A). Leave-one-out analysis showed that all the pooled estimates after omitting one study at a time were within the 95% CI of the overall estimate (Supplementary 6-B). Three outliers [49, 54, 63] were then identified by the three algorithms (Supplementary 6-C), and the meta-analysis without these outliers still showed significant heterogeneity (*I*^2^ = 71%, *P* < 0.01). The subsequent Baujat plot showed 2 studies [57, 64] lying at the right side of the plot (Supplementary 6-D). We then reperformed the meta-analysis with these 2 outliers excluded, and the fixed-effects model showed that the pooled estimate of age for G11778A LHON patients at study enrolment was 35.3 years (33.2 years and 37.3 years) (Figure 6), with a low level of heterogeneity (*I*^2^ = 19%, *P* = 0.29).

### 3.6. Onset Age of G11778A LHON Patients

**Figure 7:**
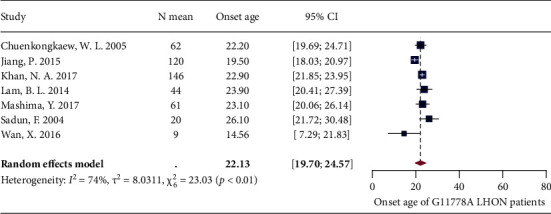
Forest plot of patient onset age.

A total of 12 studies reported the patient onset age. Significant heterogeneity was detected after pooling these studies using meta-analysis (*I*^2^ = 88%, *P* < 0.01) (Supplementary 7-A). Leave-one-out analysis showed that all the pooled estimates after omitting one study at a time were within the 95% CI of the overall estimate (Supplementary 7-B). The three algorithms were first applied, and 4 potential outliers [56, 64–66] were identified (Supplementary 7-C). The meta-analysis was then reperformed, but the significant heterogeneity still existed (*I*^2^ = 84%, *P* < 0.01). The subsequent Baujat plot identified another outlier [58] (Supplementary 7-D). We then reperformed the meta-analysis with this outlier excluded, and a decline in heterogeneity was shown (*I*^2^ = 74%, *P* < 0.01). The random-effects model showed that the pooled estimate of onset age among G11778A LHON patients was 22.1 years (19.7 years and 24.6 years) (Figure 7).

Our systematic review also identified a subgroup of G11778A LHON patients with early disease onset (onset age ≤ 18 years). Data of VA were available for 114 patients from 7 original studies covering population from China, South Korea, the UK, and Brazil (Table S3). We merged all patient cohorts from these studies to generate a patient subgroup with early disease onset for further analysis of patient characteristics. The mean onset age of this subgroup was 12.5 (SD = 4.2) years, and the male patients accounted for more than 80%. The mean VA of left eyes among these patients was 1.3 (SD = 0.6) LogMAR, while for right eyes, it was 1.3 (SD = 0.7) LogMAR. The number of eyes with VA ≤ 0.30 LogMAR was 15/220 (6.8%) whereas there were 107/220 (48.6%) eyes with a VA > 1.30 LogMAR.

### 3.7. Disease Progression

Before the onset of LHON, subclinical abnormalities were already observed in asymptomatic G11778A mutation carriers. In a study conducted in the United States of America (USA), a slight visual loss and a progressive decrease in pattern electroretinogram amplitude over time were detected in unaffected G11778A carriers [51]. In another study conducted in Brazil, subclinical abnormalities such as retinal nerve fibre layer thickening of the arcuate bundles were observed in unaffected G11778A carriers as well [47].

**Figure 8:**
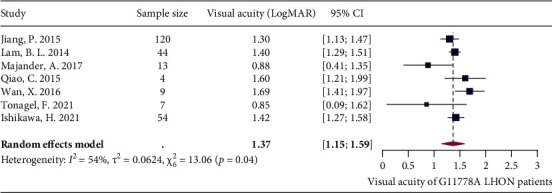
Forest plot of visual acuity (LogMAR).

For patients who were already affected by LHON at their study enrolment, the mean disease duration ranged from 6.6 (SD = 11.1) years to 23.0 (SD = 20.0) years [44, 49, 56, 67]. A total of 10 studies reported VA at baseline. In order to perform the meta-analysis, VA measured using decimal in 5 studies [58, 59, 63, 65, 67] and VA measured using the Early Treatment Diabetic Retinopathy Study (ETDRS) chart in 1 study [44] were transformed to VA expressed in LogMAR [68]. A significant heterogeneity was shown after the meta-analysis was performed (*I*^2^ = 84%, *P* < 0.01) (Supplementary 8-A). Leave-one-out analysis showed that all the pooled estimates after omitting one study at a time were within the 95% CI of the overall estimate (Supplementary 8-B). The heterogeneity remained significant after the outlier [49] identified by the three algorithms was excluded (*I*^2^ = 72%, *P* < 0.01) (Supplementary 8-C). After excluding another two outliers [65, 69] identified by the Baujat plot (Supplementary 8-D), the heterogeneity continued to decline (*I*^2^ = 54%, *P* = 0.04), and the random-effects model showed that the pooled estimate of VA for G11778A LHON patients was 1.4 LogMAR (1.2 LogMAR and 1.6 LogMAR) (Figure 8).

Regarding the natural history of LHON, in a prospective observational study conducted by Lam et al. [44] in the USA from 2008 to 2012, all 44 G11778A LHON patients included in the study were followed for 6 to 36 months with mean disease duration of 8.1 (SD = 10.5) years at enrolment. For those who had a disease duration less or equal to 1 year at enrolment, mean VA was 1.2 (SD = 0.4) LogMAR, and average mean deviation of visual field was -20.6 (SD = 10.1) dB at baseline. For those who already had a disease duration of more than 1 year at enrolment, mean VA was 1.5 (SD = 0.3) LogMAR), and average mean deviation of visual field was -26.0 (SD = 7.9) dB at baseline. During the follow-up, 13 eyes of 8 patients (18%) improved, 7 eyes of 6 patients (14%) worsened, and 68 eyes of 38 patients (86%) were stable (15 ETDRS letters (1.4 LogMAR) or more gain was defined as improved from baseline, while 15 ETDRS letters or more loss was defined as worsened from baseline). For worsened eyes, all worsening occurred within 1 year after the onset of VA loss and reached the lowest levels about 1 year after the onset of symptoms. The mean deviation was found to have worsened correspondingly with the VA in worsened eyes. For patients with stable eyes, median time since the acuity loss onset was 5.5 years.

Differences in disease onset and progression between two eyes were also reported in several studies. In a prospective cohort study conducted in Brazil [49] with 20 G11778A LHON patients included, the mean interval from disease onset to clinical examination at enrolment was 18.8 years and the mean best corrected visual acuity (BCVA) was 2.0 ± 0.6 LogMAR (female: 2.2 and male: 2.0). There were 75% (15/20) of patients with bilateral simultaneous onset, while the mean interval of onset between the first affected eye and the fellow eye was 16.8 weeks for the remaining 5 cases, which was consistent with the median interval of 2 months as reported by another prospective observational study in the USA [44]. According to a prospective observational study [70] conducted in China, among 33 patients with different disease onset between eyes, the time interval of disease onset between eyes ranged from 1 month to 33 months. This study also found that the difference in visual function between eyes decreased along with the disease progression. The mean of difference in visual field between eyes decreased from 41.0% in visual field index (VFI) and 12.1 dB in mean deviation (MD) for patients with disease duration less than 1 month to 10.6% in VFI and 3.6 dB in MD for patients with disease duration between 12 and 24 months.

### 3.8. Visual Recovery

**Figure 9:**
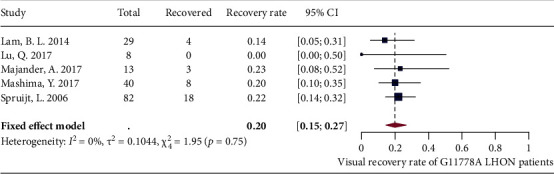
Forest plot of spontaneous visual recovery rate.

A total of 8 studies reported the spontaneous visual recovery rate of G11778A LHON patients, while inconsistent definitions of spontaneous visual recovery were used across studies, and there were also differences in onset age and time from onset to evaluation in different patient cohorts (Table S2). Significant heterogeneity was detected after pooling the studies via meta-analysis (*I*^2^ = 52%, *P* = 0.04) (Supplementary 9-A). Leave-one-out analysis showed that all the pooled estimates after omitting one study at a time were within the 95% CI of the overall estimate (Supplementary 9-B). After excluding two outliers [49, 71] identified by the three algorithms (Supplementary 9-C) and another outlier [72] identified by the Baujat plot (Supplementary 9-D), the fixed-effects model showed that the pooled estimate of spontaneous recovery rate (in either 1 eye) for G11778A LHON patients was 20% (15% and 27%), with a low level of heterogeneity (*I*^2^ = 0%, *P* = 0.75) (Figure 9).

Regarding the time to recovery, a study conducted in the UK reported that the mean time to spontaneous visual recovery was 27 months [67], which was consistent with the median time of 27.5 months as reported from another study conducted in the USA [44].

### 3.9. Risk Factors

#### 3.9.1. Gender Bias

According to a study conducted in India with 543 individuals carrying G11778A enrolled, the proportion of carriers who developed visual loss was much higher in males (37.9%) than that in females (13.7%) [73]. Another study from China with a large sample size of 1859 also demonstrated that gender was the most significant factor for influencing the clinical manifestation of LHON (3.48-fold) in Chinese families [60].

#### 3.9.2. Proportion of Mutant G11778A

A study investigating the association between the proportion of mutant G11778A mtDNA and clinical manifestation in a Thai population showed that this proportion was correlated with likelihood of developing visual loss. Individuals who had mutant G11778A mtDNA of more than 75% were more likely to develop blindness than those with mutant mtDNA of less than 75% (odds ratio = 6.41) [74].

#### 3.9.3. Haplogroups

A study with 1859 G11778A carriers from 182 Chinese families demonstrated that the haplogroup M7b1′2 could significantly increase the risk of visual loss, while M8a might have a protective effect [60]. In addition, two unrelated families with LHON patients carrying G11778A mutation in China were studied by Qiao et al. Results from this study suggested that secondary mutations including T3394C and T14502C were potential polymorphism acting synergistically with G11778A mutation to induce visual loss and H2a2a1 haplogroup could be a protective factor on phenotypic LHON manifestation [58]. Another study reported markedly and statistically lower prevalence of hypertension, stroke, and cerebral vascular accident for G11778A carriers and affected patients than those of off-pedigree controls within a LHON pedigree, suggesting potential protective effect against cardiovascular diseases of the maternal lineage featured by presence of homoplasmic polymorphisms at positions 10398, 4216, and 13708, in the ND3, ND1, and ND5 subunit genes of mitochondrial complex I, respectively [46].

#### 3.9.4. Optic Nerve Head

The association of optic nerve head morphology with development and prognosis of G11778A LHON was studied by Ramos et al. on pedigrees from Brazilian and Italian. This study suggested that a larger optic nerve head size could be a protective factor for G11778A LHON [72].

#### 3.9.5. Environmental Factors

Results from a study conducted in Brazil showed that the affected G11778A LHON patients were more likely to smoke while having a higher toxic exposure. Alcohol consumption was also much more common in affected patients.

### 3.10. Treatment

#### 3.10.1. Idebenone

Idebenone is a synthetic, short-chain analogue of coenzyme Q, which has been reported to be a potential agent to treat LHON, but the treatment effectiveness is controversial. Zhao et al. reported significant improvement in VA after 3 and 6 months of treatment with 900 mg/d idebenone with an average change from 1.4 ± 0.9 LogMAR at baseline to 1.0 ± 0.7 LogMAR after 3 months and 0.8 ± 0.6 LogMAR after 6 months [75]. In a prospective and noncomparative study [56] conducted in Japan, 33.3% (17/51) of the patients who received 900 mg/d idebenone showed significant improvement in BCVA (>0.2 LogMAR change in at least 1 eye at 48 weeks compared with baseline data), and it was also found that early intervention was associated with significantly higher improvement rate. While for visual fields (VFs) in MD values measured by Humphrey Field Analyzer (HFA) and critical fusion frequency (CFF) values which are defined as the frequency at which a flickering light is perceived as continuous, no significant improvement (>20% improvement compared with baseline) was observed at week 48 [56]. In a phase II multicentre, double-blind, randomized, placebo-controlled trial of idebenone conducted among 85 LHON patients [76], the difference between the treatment and placebo group in the best recovery of VA between baseline and week 24 did not meet statistical significance, while significant difference was observed in the subgroup of patients with discordant VA at baseline. Despite studies suggesting potential benefits of idebenone in the treatment of LHON, several studies reported nonsignificant effect of idebenone instead. Mashima et al. compared the final VA between patients who received 90 mg/day idebenone with those who did not receive it, and no significant difference between these two patient groups was found [61]. A prospective observational study conducted in the USA also compared the proportions of patients with visual recovery in patients who received idebenone and in those who did not receive it, while no significant difference between the two groups was observed (*P* = 0.41) [44].

#### 3.10.2. Gene Therapy

In an investigator-initiated trial conducted in China with 9 G11778A LHON patients included, all participants received recombinant adeno-associated virus 2 carrying ND4 (rAAV2-ND4) by intravitreal injection to one eye (except one patient who received injection in both eyes). After 9 months of follow-up, 6 patients showed improved VA by at least 0.3 LogMAR in the injected eyes. Local or systemic adverse events related to the vector were not found in all 9 patients. The findings from this study suggested that single-dose intravitreal rAAV2-ND4 injection can be safe and effective among patients with G11778A mutation [50]. Eight out of 9 patients in this trial were followed up for 75 to 90 months, during which 6 patients maintained clinically significant improvement in BCVA (≥0.3 LogMAR), while no systemic or ocular adverse event was detected, indicating long-term safety and durability of the rAAV2-ND4 treatment [77]. Another two multicentre, randomized, sham-controlled phase 3 clinical trials conducted in France, Germany, Italy, the UK, and the USA, REVERSE (NCT02652780) and RESCUE (NCT02652767) also evaluated the efficacy of rAAV2-ND4 among LHON patients [78, 79]. In REVERSE study, rAAV2-ND4-treated eyes showed a significant mean BCVA improvement of -0.30 LogMAR and sham-treated eyes showed a mean BCVA improvement of -0.26 LogMAR at week 96 among 37 patients with duration of vision loss from 6 to 12 months [78]. While in RESCUE study, a profound gain of +10 ETDRS letters equivalent in treated eyes and +9 ETDRS letters equivalent in untreated eyes was observed from week 48 to week 96 among 38 patients with duration of vision loss ≤ 6 months [79]. The unexpected bilateral improvement in vision may be caused by transfer of viral vector DNA from the treated eyes to the contralateral sham-treated eye as suggested by a nonhuman primate study.

In 2016, the Bascom Palmer Eye Institute in the USA published the initial results of a gene therapy trial with 5 G11778A LHON patients enrolled (NCT02161380). All participants received a single intravitreal injection of self-complementary adeno-associated vector- (scAAV2-) (Y444, 500, 730F) P1ND4v2. Three months after enrolment, two participants showed improvements in VA, one showed an increase from hand movements at baseline to 7 ETDRS letters at 3 months postbaseline, and one showed an increase of 15 letters by 3 months postbaseline. No serious adverse event was observed during the follow-up period [64]. In 2017, the Bascom Palmer Eye Institute published the visual results of 14 G11778A LHON patients who received the same gene therapy. For 12 of 14 patients who had developed bilateral visual loss, the average improvement in VA at 12 months post injection from baseline was 0.24 LogMAR for injected eyes and 0.09 LogMAR for the fellow eyes. Thus, this gene therapy showed effect on VA improvement, and no safety concern was raised for this therapy [66].

## 4. Discussion

LHON is one of the most common inherited mitochondrial disorders caused by mutations in the mtDNA with progressive vision loss [1]. Three primary mtDNA mutations for LHON are G11778A, G3460A, and T14484C, among which G11778A is the most prevalent. According to our study, G11778A accounted 73% (67% and 79%) of the three primary mutations for LHON. Although this mtDNA mutation is the primary etiological factor of LHON, not all individuals who carry G11778A mutation in their mtDNAs will eventually develop visual loss. The penetrance of LHON is variable and is associated with a number of factors such as gender, mutant G11778A proportion, haplogroups, optic nerve head morphology, and several environmental triggers (e.g., smoking and drinking).

The acute stage of LHON is marked by a rapid visual loss within six months after first symptom and a progressively evolving central scotoma, as observed from studies with G11778A carriers converting from asymptomatic carriers to affected patients [47, 51]. The time interval of onset between the first affected eye and the fellow eye is generally within 3 years and could be as short as 1 month [44, 49, 70]. The onset of LHON could occur in a long time period from the first to the eighth decade of life, while the peak age of onset of visual loss among LHON carriers varies from 20 to 30 years old [3, 80]. In our study, the mean onset age of LHON in patients carrying G11778A mutation was 22.1 years (19.7 years and 24.6 years), which is consistent with the previous findings [9, 81]. Our study also identified a male predominance for G11778A LHON, with males accounting for 77% (76% and 79%) of all G11778A LHON cases.

LHON patients are usually affected by monocular or binocular visual impairment, which is one of the common clinical features of many eye diseases. For adolescent patients with sudden binocular vision loss, the possibility of LHON should be considered when other common eye diseases causing vision loss are excluded, such as keratoconus and optic neuritis. LHON patients display normal pupil reflex to light, slight hyperemia of the optic disc in both eyes, and dark spot in the centre of the binocular field. For the mtDNA mutation detection related to LHON, it is suggested that in addition to the detection of common mutation sites, other pathogenic gene sites should also be examined to avoid omissions. Very few patients of the disease may also show dyspraxia, tremors, heart conduction defects, muscle weakness, numbness, poor coordination, and other symptoms.

Overall, the visual prognosis of G11778A LHON is poor. The pooled VA identified in our study was 1.4 LogMAR (1.2 LogMAR and 1.6 LogMAR) Spontaneous visual recovery was rare and limitedly observed for G11778A LHON patients with a spontaneous visual recovery rate of 20% (15% and 27%) identified in our study. Most patients will remain visually impaired for the rest of their lives, according to our study. The mean disease duration for G11778A LHON patients ranged from 6.6 (SD = 11.1) years in a Japanese patient cohort to 23.0 (SD = 20.0) years in a UK pediatric patient cohort [44, 49, 56, 67]. In our study, we also performed a subgroup analysis within G11778A LHON patients who had early disease onset at 18 years old or younger. Male predominance was found in this subgroup with male patients accounting for more than 80%. Visual acuity for this subgroup, the mean VA of both eyes as 1.3 LogMAR, was poorer than the mean VA of 0.7 LogMAR reported from a previous study investigating childhood-onset LHON [67]. Since early onset is a positive prognostic factor of LHON [9], a possible explanation for this difference is that the study of Majander et al. included patients with onset age of 12 years or younger, while our study included patients with onset age of 18 years or younger for this subgroup analysis. The mean onset age identified in our study was 12.5 years, while in the study of Majander et al., it was 8.3 years.

LHON is a debilitating disease that significantly impacts patients and their families with no effective treatment available. Effectiveness of coenzyme Q10 [82, 83] and idebenone in improving visual acuity of G11778A LHON patients shown in previous studies [44, 61, 84] was inconclusive. However, in recent years, the exploration of gene therapy for treating LHON patients has brought hope for LHON patients. Preliminary results provided by several clinical trials have shown that gene therapies such as rAAV2-ND4 and scAAV2- (Y444, 500, 730F) P1ND4v2 were well tolerated and improved visual outcomes of G11778A LHON patients [50, 64, 66, 77–79]. In the future, more in-depth basic research is needed to guide clinical practice to provide more effective and safe treatment for LHON patients.

These systematic review and meta-analysis comprehensively summarized evidence regarding the epidemiology, disease onset and progression, visual recovery, risk factors, and treatment options of LHON patients with G11778A mutation. The main limitation of this study is the various data sources and uneven sample size from included studies due to the specificity of LHON as a rare disease, leading to significant heterogeneity of the pooled results in our meta-analysis. This limitation has been addressed at least in part by using algorithms as well as generating the Baujat plot to identify and exclude potential outliers among included studies, which could strengthen the robustness of the results. Another limitation is regarding the VA value conversion. We have used sample-based formulae to transform study-level VA results to LogMAR format before conducting the meta-analysis. Assumption of LogMAR normal distribution in the population from which the study sample was drawn is made, which cannot be adequately verified. Also, the definition of spontaneous visual recovery and significant improvement in visual acuity varied from study to study, so the pooled recovery rate needs to be interpreted with caution, and the effectiveness of available or potential treatments remains inconclusive.

## 5. Conclusions

In summary, LHON is a progressive, male-predominant disease with early disease onset and usually causes devastating central visual loss to patients. G11778A mutation is a prevalent mitochondrial genetic mutation accounting for over half of the overall LHON cases with three primary mutations. With the gradual understanding of the disease, its characteristics, clinical manifestations, accompanying symptoms, and so on are gradually clear, but there is still a problem that cannot be solved, its treatment. Spontaneous visual recovery is rare and limited in G11778A LHON patients, and no effective treatment is currently available. Therefore, there is an urgent need to explore new treatment options for LHON patients. Idebenone has been shown to be effective in the treatment of LHON, but only in patients with a short period of onset. Preliminary results from several early-phase clinical trials have shown the effectiveness and safety of gene therapies, which brought hope for therapeutic breakthroughs in LHON in the near future.

## Data Availability

All data is available.

## Supplementary Materials

Supplementary 1 S. Table 1: characteristics of the included studies.

Supplementary 2 S. Table 2: data summary of the previously published G11778A LHON patients with report of spontaneous visual recovery rate.

Supplementary 3 S. Table 3: data summary of the previously published G11778A LHON patients with onset of visual loss less than 18 years of age.

Supplementary 4 S. Figure 1: systematic literature review protocol.

Supplementary 5 S. Figure 2: evaluation questions for title and abstract screening.

Supplementary 6 S. Figure 3: risk of bias assessment.

Supplementary 7 S. Figure 4-A: forest plot of G11778A mutation rate among 3 primary LHON mutations. S. Figure 4-B: leave-one-out analysis of studies reporting G11778A mutation rate among 3 primary LHON mutations. S. Figure 4-C: potential outliers identified from *K*-means clustering, DBSCAN, and Gaussian mixture models in studies reporting G11778A mutation rate among 3 primary LHON mutations. S. Figure 4-D: the Baujat plot of the influence of remaining studies reporting G11778A mutation rate among 3 primary LHON mutations after excluding potential outliers identified previously by *K*-means clustering, DBSCAN, and Gaussian mixture models.

Supplementary 8 S. Figure 5-A: forest plot of male ratio of G11778A LHON patients. S. Figure 5-B: leave-one-out analysis of studies reporting male ratio of G11778A LHON patients. S. Figure 5-C: potential outliers identified from *K*-means clustering, DBSCAN, and Gaussian mixture models in studies reporting male ratio of G11778A LHON patients.

Supplementary 9 S. Figure 6-A: forest plot of age of G11778A LHON patients. S. Figure 6-B: leave-one-out analysis of studies reporting age of G11778A LHON patients. S. Figure 6-C: potential outliers identified from *K*-means clustering, DBSCAN, and Gaussian mixture models in studies reporting age of G11778A LHON patients. S. Figure 6-D: the Baujat plot of the influence of remaining studies reporting age of G11778A LHON patients after excluding potential outliers identified previously by *K*-means clustering, DBSCAN, and Gaussian mixture models.

Supplementary 10 S. Figure 7-A: forest plot of onset age of G11778A LHON patients. S. Figure 7-B: leave-one-out analysis of studies reporting onset age of G11778A LHON patients. S. Figure 7-C: potential outliers identified from *K*-means clustering, DBSCAN, and Gaussian mixture models in studies reporting onset age of G11778A LHON patients. S. Figure 7-D: the Baujat plot of the influence of remaining studies reporting onset age of G11778A LHON patients after excluding potential outliers identified previously by *K*-means clustering, DBSCAN, and Gaussian mixture models.

Supplementary 11 S. Figure 8-A: forest plot of visual acuity of G11778A LHON patients. S. Figure 8-B: leave-one-out analysis of studies reporting visual acuity of G11778A LHON patients. S. Figure 8-C: potential outliers identified from *K*-means clustering, DBSCAN, and Gaussian mixture models in studies reporting visual acuity of G11778A LHON patients. S. Figure 8-D: the Baujat plot of the influence of remaining studies reporting visual acuity of G11778A LHON patients after excluding potential outliers identified previously by *K*-means clustering, DBSCAN, and Gaussian mixture models.

Supplementary 12 S. Figure 9-A: forest plot of visual recovery rate of G11778A LHON patients. S. Figure 9-B: leave-one-out analysis of studies reporting visual recovery rate of G11778A LHON patients. S. Figure 9-C: potential outliers identified from *K*-means clustering, DBSCAN, and Gaussian mixture models in studies reporting visual recovery rate of G11778A LHON patients. S. Figure 9-D: the Baujat plot of the influence of remaining studies reporting visual recovery rate of G11778A LHON patients after excluding potential outliers identified previously by *K*-means clustering, DBSCAN, and Gaussian mixture models.
