# Supplementary material for: Leber's Hereditary Optic Neuropathy with Mitochondrial DNA Mutation G11778A: A Systematic Literature Review and Meta-Analysis
Source: Biomed Res Int. 2023 Jan 24;2023:1107866. doi: 10.1155/2023/1107866 (PMC9893526; doi:10.1155/2023/1107866)
Supplement: Supplementary 1 — S. Table 1: characteristics of the included studies. [file 1107866.f1.pdf]

Table S1: Characteristics of included studies

Number	Author Year	Geography	Number of LHON Patients	Number of LHON Patients with G11778A	Proportion of G11778A in LHON	Age of G11778A LHON Patients	Gender Ratio in G11778A LHON Patients (M/F)
1	Qiao et al. 2015 [1]	China	4	4	/	mean: 40.3 ± 30.7 median:37 range: 12-75	1/3
2	Lu et al. 2017 [2]	China	8	8	/	mean: 45.8 median: 36.5 range: 27-88	4/4
3	Wan et al. 2016 [3]	China	9	9	/	mean: 19.2 ± 11.6 median: 17 range: 9-46	7/2
4	Du et al. 2011 [4]	China	18	10	55.6%	/	/

5	Cui et al. 2013 [5]	China	50	38	76%	/	/
6	Jiang et al. 2015 [6]	China	1281	453	35.4%	/	108/12 (analyzed)
7	Ji et al. 2008 [7]	China	619	619	/	/	436/183
8	A.A. Sadun et al. 2006 [8]*	Brazil	75	2	/	patient 1: 12<br>patient 2: 44	2/0
9	A.A. Sadun et al. 2002 [9]	Brazil	20	20	/	mean:<br>46.8 ± 17.2	17/3
10	A.A. Sadun et al. 2003 [10]	Brazil	20	20	/	/	17/3
11	F. Sadun et al. 2004 [11]	Brazil	20	20	/	mean:<br>47 ± 17<br>range: 14-84	17/3
12	Ramos Cdo et al. 2009 [12]	Brazil/<br>Italy	71	45	63.4%	/	/
13	Guy et al. 2014 [13]**	US	45	1	/	19	1/0
14	Feuer et al. 2016 [14]	US	5	5	/	mean: 44.2	4/1

						range:35-55	
15	Guy et al. 2017 [15]	US	14	14	/	mean: 33.8<br>median: 30<br>range: 19-56	10/4
16	Lam et al. 2014 [16]	US	44	44	/	mean: 32.1±13<br>range: 10-61	35/9
17	Ueda et al. 2017 [17]	Japan	44	38	86.4%	/	/
18	Mashima et al. 2017 [18]	Japan	61	61	/	/	56/5
19	Mishra et al. 2017 [19]	India	40	11	27.5%	mean:<br>19.6 ± 8.8<br>median:16<br>range: 11-42	11/0
20	Khan et al. 2017 [20]	India	146	146	/	/	112/34
21	Majander et al. 2017 [21]	UK	27	13	48.1%	/	10/3
22	Dimitriadis et al. 2014 [22]	Germany	253	167	66%	/	13/3<br>(late onset)

23	Spruijt et al. 2006 [23]	Netherland	351	145	41.3%	/	124/21
24	Kirkman et al. 2009 [24]	UK/ Netherlands /Germany	196	132	67.3%	/	/
25	Klopstock et al. 2011 [25]	UK/Canada /Germany	85	57	67.1%	/	/
26	Kim et al. 2003 [26]	Korea	82	46	56.1%	/	/
27	Chuenkongkaew et al. 2005 [27]	Thailand	62	62	/	mean: 37.3 ± 12.9 range: 8-68	47/15
28	Gowri et al. 2020 [28]	India	55	23	41.8%	/	/
29	Tonagel et al. 2021 [29]	Germany	33	18	54.5%	/	7/0 (analyzed)
30	Yu-Wai-Man et al. 2020 [30]	France/Germany/Italy/	37	37	/	/	29/8

		UK/US					
31	Ishikawa et al. 2021 [31]	Japan	57	54	94.7%	mean: 35.3 ± 17.9 median: 32.3 range: 11.2-75	49/5
32	Marotta et al. 2020 [32]	Australia	76	52	68.4%	mean: 26.4	40/12
33	Li et al. 2020 [33]	China	132	84	63.6%	mean: 28.7 ± 16.0 median: 25.5 range: 0.8-66	56/28
34	Ahn et al. 2020 [34]	Korea	64	38	59.4%	/	10/2 (early onset)
35	Poincenot et al. 2020 [35]	Global	1512	1044	69.0%	/	795/249
36	Yang et al. 2020 [36]	China	9	9	/	mean: 19.2 ± 11.6	7/2

						median: 17 range: 9-46	
37	Cui et al. 2019 [37]	China	73	73	/	/	49/6 (analyzed)
38	Dokrungkoon et al. 2019 [38]	Thailand	13	10	76.9%	/	/
39	Liu et al. 2019 [39]	China	80	80	/	/	68/12
40	Newman et al. 2021 [40]	France/Germany/Italy/UK/US	39	39	/	mean: 36.8 ± 15.4 range: 15-69	31/7 (analyzed)
41	Zhao et al. 2020 [41]	China	33	29	87.9%	/	/

LHON = Leber hereditary optic neuropathy.

* In the study of Sadun et al. 2006, 2 out of 75 individuals converted from G11778A carriers to LHON-affected patients.

** In the study of Guy et al. 2014, 1 out of 45 individuals converted from G11778A carrier to LHON-affected patient.

Reference

1. Qiao C, Wei T, Hu B, Peng C, Qiu X, Wei L, Yan M. Two families with Leber's hereditary optic neuropathy carrying G11778A and T14502C mutations with haplogroup H2a2a1 in mitochondrial DNA. *Mol Med Rep* 2015; **12**: 3067-72.
2. Lu Q, Guo Y, Yi J, Deng X, Yang Z, Yuan X, Deng H. Identification of an ND4 Mutation in Leber Hereditary Optic Neuropathy. *Optom Vis Sci* 2017; **94**: 1090-4.
3. Wan X, Pei H, Zhao MJ, Yang S, Hu WK, He H, Ma SQ, Zhang G, Dong XY, Chen C, Wang DW, Li B. Efficacy and Safety of rAAV2-ND4 Treatment for Leber's Hereditary Optic Neuropathy. *Sci Rep* 2016; **6**: 21587.
4. Du WD, Chen G, Cao HM, Jin QH, Liao RF, He XC, Chen DB, Huang SR, Zhao H, Lv YM, Tang HY, Tang XF, Wang YQ, Sun S, Zhao JL, Zhang XJ. A simple oligonucleotide biochip capable of rapidly detecting known mitochondrial DNA mutations in Chinese patients with Leber's hereditary optic neuropathy (LHON). *Dis Markers* 2011; **30**: 181-90.
5. Cui G, Ding H, Xu Y, Li B, Wang DW. Applications of the method of high resolution melting analysis for diagnosis of Leber's disease and the three primary mutation spectrum of LHON in the Han Chinese population. *Gene* 2013; **512**: 108-12.
6. Jiang P, Liang M, Zhang J, Gao Y, He Z, Yu H, Zhao F, Ji Y, Liu X, Zhang M, Fu Q, Tong Y, Sun Y, Zhou X, Huang T, Qu J, Guan MX. Prevalence of Mitochondrial ND4 Mutations in 1281 Han Chinese Subjects With Leber's Hereditary Optic Neuropathy. *Invest Ophthalmol Vis Sci* 2015; **56**: 4778-88.
7. Ji Y, Zhang AM, Jia X, Zhang YP, Xiao X, Li S, Guo X, Bandelt HJ, Zhang Q, Yao YG. Mitochondrial DNA haplogroups M7b1'2 and M8a affect clinical expression of leber hereditary optic neuropathy in Chinese families with the m.11778G-->a mutation. *Am J Hum Genet* 2008; **83**: 760-8.
8. Sadun AA, Salomao SR, Berezovsky A, Sadun F, Denegri AM, Quiros PA, Chicani F, Ventura D, Barboni P, Sherman J, Sutter E, Belfort R, Jr., Carelli V. Subclinical carriers and conversions in Leber hereditary optic neuropathy: a prospective psychophysical study. *Trans Am Ophthalmol Soc* 2006; **104**: 51-61.
9. Sadun AA, Carelli V, Salomao SR, Berezovsky A, Quiros P, Sadun F, DeNegri AM, Andrade R, Schein S, Belfort R. A very large Brazilian pedigree with 11778 Leber's hereditary optic neuropathy. *Trans Am Ophthalmol Soc* 2002; **100**: 169-78; discussion 78-9.
10. Sadun AA, Carelli V, Salomao SR, Berezovsky A, Quiros PA, Sadun F, DeNegri AM, Andrade R, Moraes M, Passos A, Kjaer P, Pereira J, Valentino ML, Schein S, Belfort R. Extensive investigation of a large Brazilian pedigree of 11778/haplogroup J Leber hereditary optic neuropathy. *Am J Ophthalmol* 2003; **136**: 231-8.
11. Sadun F, De Negri AM, Carelli V, Salomao SR, Berezovsky A, Andrade R, Moraes M, Passos A, Belfort R, da Rosa AB, Quiros P, Sadun AA. Ophthalmologic findings in a large pedigree of 11778/Haplogroup J Leber hereditary optic neuropathy. *Am J Ophthalmol* 2004; **137**: 271-7.
12. Ramos Cdo V, Bellusci C, Savini G, Carbonelli M, Berezovsky A, Tamaki C, Cinoto R, Sacai PY, Moraes-Filho MN, Miura HM, Valentino ML, Iommarini L, De Negri AM, Sadun F, Cortelli P, Montagna P, Salomao SR, Sadun AA, Carelli V, Barboni P. Association of optic disc size with development and prognosis of Leber's hereditary optic neuropathy. *Invest Ophthalmol Vis Sci* 2009; **50**: 1666-74.
13. Guy J, Feuer WJ, Porciatti V, Schiffman J, Abukhalil F, Vandenbroucke R, Rosa PR, Lam BL. Retinal

ganglion cell dysfunction in asymptomatic G11778A: Leber hereditary optic neuropathy. *Invest Ophthalmol Vis Sci* 2014; **55**: 841-8.

14. Feuer WJ, Schiffman JC, Davis JL, Porciatti V, Gonzalez P, Koilkonda RD, Yuan H, Lalwani A, Lam BL, Guy J. Gene Therapy for Leber Hereditary Optic Neuropathy: Initial Results. *Ophthalmology* 2016; **123**: 558-70.
15. Guy J, Feuer WJ, Davis JL, Porciatti V, Gonzalez PJ, Koilkonda RD, Yuan H, Hauswirth WW, Lam BL. Gene Therapy for Leber Hereditary Optic Neuropathy: Low- and Medium-Dose Visual Results. *Ophthalmology* 2017; **124**: 1621-34.
16. Lam BL, Feuer WJ, Schiffman JC, Porciatti V, Vandenbroucke R, Rosa PR, Gregori G, Guy J. Trial end points and natural history in patients with G11778A Leber hereditary optic neuropathy : preparation for gene therapy clinical trial. *JAMA Ophthalmol* 2014; **132**: 428-36.
17. Ueda K, Morizane Y, Shiraga F, Shikishima K, Ishikawa H, Wakakura M, Nakamura M. Nationwide epidemiological survey of Leber hereditary optic neuropathy in Japan. *J Epidemiol* 2017; **27**: 447-50.
18. Mashima Y, Kigasawa K, Shinoda K, Wakakura M, Oguchi Y. Visual prognosis better in eyes with less severe reduction of visual acuity one year after onset of Leber hereditary optic neuropathy caused by the 11,778 mutation. *BMC Ophthalmol* 2017; **17**: 192.
19. Mishra A, Devi S, Saxena R, Gupta N, Kabra M, Chowdhury MR. Frequency of primary mutations of Leber's hereditary optic neuropathy patients in North Indian population. *Indian J Ophthalmol* 2017; **65**: 1156-60.
20. Khan NA, Govindaraj P, Soumittra N, Sharma S, Srilekha S, Ambika S, Vanniarajan A, Meena AK, Uppin MS, Sundaram C. Leber's Hereditary Optic Neuropathy–Specific Mutation m. 11778G> A Exists on Diverse Mitochondrial Haplogroups in India. *Investigative ophthalmology & visual science* 2017; **58**: 3923-30.
21. Majander A, Bowman R, Poulton J, Antcliff RJ, Reddy MA, Michaelides M, Webster AR, Chinnery PF, Votruba M, Moore AT, Yu-Wai-Man P. Childhood-onset Leber hereditary optic neuropathy. *Br J Ophthalmol* 2017; **101**: 1505-9.
22. Dimitriadis K, Leonhardt M, Yu-Wai-Man P, Kirkman MA, Korsten A, De Coo IF, Chinnery PF, Klopstock T. Leber's hereditary optic neuropathy with late disease onset: clinical and molecular characteristics of 20 patients. *Orphanet J Rare Dis* 2014; **9**: 158.
23. Spruijt L, Kolbach DN, de Coo RF, Plomp AS, Bauer NJ, Smeets HJ, de Die-Smulders CE. Influence of mutation type on clinical expression of Leber hereditary optic neuropathy. *Am J Ophthalmol* 2006; **141**: 676-82.
24. Kirkman MA, Korsten A, Leonhardt M, Dimitriadis K, De Coo IF, Klopstock T, Griffiths PG, Hudson G, Chinnery PF, Yu-Wai-Man P. Quality of life in patients with leber hereditary optic neuropathy. *Invest Ophthalmol Vis Sci* 2009; **50**: 3112-5.
25. Klopstock T, Yu-Wai-Man P, Dimitriadis K, Rouleau J, Heck S, Bailie M, Atawan A, Chattopadhyay S, Schubert M, Garip A. A randomized placebo-controlled trial of idebenone in Leber's hereditary optic neuropathy. *Brain* 2011; **134**: 2677-86.
26. Kim JY, Hwang JM, Chang BL, Park SS. Spectrum of the mitochondrial DNA mutations of Leber's hereditary optic neuropathy in Koreans. *J Neurol* 2003; **250**: 278-81.
27. Chuenkongkaew WL, Suphavilai R, Vaeusorn L, Phasukkijwatana N, Lertrit P, Suktitipat B. Proportion of 11778 mutant mitochondrial DNA and clinical expression in a thai population with leber hereditary optic neuropathy. *J Neuroophthalmol* 2005; **25**: 173-5.

28. Gowri P, Kumar SM, Vanniarajan A, Bharanidharan D, Sundaresan P. A hospital-based five-year prospective study on the prevalence of Leber's hereditary optic neuropathy with genetic confirmation. *Mol Vis* 2020; **26**: 789–96.
29. Tonagel FA-O, Wilhelm H, Richter P, Kelbsch C. Leber's hereditary optic neuropathy: course of disease in consideration of idebenone treatment and type of mutation. *Graefes Arch Clin Exp Ophthalmol* 2021; **259**: 1009-13.
30. Yu-Wai-Man P, Newman NJ, Carelli V, et al. Bilateral visual improvement with unilateral gene therapy injection for Leber hereditary optic neuropathy. *Sci Transl Med* 2020; **12**: eaaz7423.
31. Ishikawa HA-O, Masuda Y, Ishikawa H, Shikisima K, Goseki T, Kezuka T, Terao M, Miyazaki A, Matsumoto K, Nishikawa H, Gomi F, Mimura O. Characteristics of Japanese patients with Leber's hereditary optic neuropathy and idebenone trial: a prospective, interventional, non-comparative study. *Jpn J Ophthalmol* 2021; **65**: 133-42.
32. Marotta R, Chin J, Chiotis M, Shuey N, Collins SJ. Long-term screening for primary mitochondrial DNA variants associated with Leber hereditary optic neuropathy: incidence, penetrance and clinical features. *Mitochondrion* 2020; **54**: 128-32.
33. Li JK, Li W, Gao FJ, Qu SF, Hu FY, Zhang SH, Li LL, Wang ZW, Qiu Y, Wang LS, Huang J, Wu JH, Chen F. Mutation Screening of mtDNA Combined Targeted Exon Sequencing in a Cohort With Suspected Hereditary Optic Neuropathy. *Transl Vis Sci Technol* 2020; **9**: 11.
34. Ahn YJ, Park Y, Shin SY, Chae H, Kim M, Park SA-O. Genotypic and phenotypic characteristics of Korean children with childhood-onset Leber's hereditary optic neuropathy. *Graefes Arch Clin Exp Ophthalmol* 2020; **258**: 2283-90.
35. Poincenot L, Pearson AL, Karanjia R. Demographics of a Large International Population of Patients Affected by Leber's Hereditary Optic Neuropathy. *Ophthalmology* 2020; **127**: 679-88.
36. Yang S, Chen C Fau - Yuan J-J, Yuan Jj Fau - Wang S-S, Wang Ss Fau - Wan X, Wan X Fau - He H, He H Fau - Ma S-Q, Ma Sq Fau - Li B, Li B. Multilocus Mitochondrial Mutations Do Not Directly Affect the Efficacy of Gene Therapy for Leber Hereditary Optic Neuropathy. *J Neuroophthalmol* 2020; **40**: 22-9.
37. Cui S, Jiang H, Peng J, Wang J, Zhang X. Evaluation of Vision-Related Quality of Life in Chinese Patients With Leber Hereditary Optic Neuropathy and the G11778A Mutation. *J Neuroophthalmol* 2019; **39**: 56-9.
38. Dokrungkoon T, Onsod P, Areesirisuk P, Rerkamnuaychoke B, Vanikieti K, Chareonsirisuthigul T. Performance of the MLPA technique for detecting common mutations in Leber hereditary optic neuropathy. *Mitochondrial DNA A DNA Mapp Seq Anal* 2019; **30**: 819-24.
39. Liu Z, Fang F, Ding C, Zhang W, Li J, Yang X, Wang X, Wu Y, Wang H, Liu L, Han T, Wang X, Chen C, Lyu J, Wu H. [Diagnosis of mitochondrial disorders in children with next generation sequencing]. *Zhonghua Er Ke Za Zhi* 2015; **53**: 747-53.
40. Newman NJ, Yu-Wai-Man P, Carelli V, et al. Efficacy and Safety of Intravitreal Gene Therapy for Leber Hereditary Optic Neuropathy Treated within 6 Months of Disease Onset. *Ophthalmology* 2021; **128**: 649-60.
41. Zhao X, Zhang Y, Lu LA-O, Yang H. Therapeutic Effects of Idebenone on Leber Hereditary Optic Neuropathy. *Current eye research* 2020; **45**: 1315-23.
