# Supplementary material for: Leber's Hereditary Optic Neuropathy with Mitochondrial DNA Mutation G11778A: A Systematic Literature Review and Meta-Analysis
Source: Biomed Res Int. 2023 Jan 24;2023:1107866. doi: 10.1155/2023/1107866 (PMC9893526; doi:10.1155/2023/1107866)
Supplement: Supplementary 2 — S. Table 2: data summary of the previously published G11778A LHON patients with report of spontaneous visual recovery rate. [file 1107866.f2.pdf]

Table S2. Data summary of previously published G11778A LHON patients with report of spontaneous visual recovery rate.

Author	Symptomatic G11778A Patients (n)	Age at enrolment (years)	Onset age (years)	Time from onset	Definition of visual recovery	Recovery rate
Lam, B. L. et al. 2014 [1]	44<br>(29 no treatment, 15 on idebenone)	Mean: 32.1<br>SD: 13.0<br>Range: 10-61	Mean: 23.9<br>SD: 11.8<br>Range: 4-61	≤ 72 months	Improvement of visual acuity by 15 letters in either eye from baseline	13.79% (4/29)
Lu, Q et al. 2017 [2]	8	Mean: 45.8<br>SD: 22.7<br>Median: 36.5<br>Range: 27-88	Mean: 24.3<br>SD: 20.1<br>Median: 14.5<br>Range: 10-64	Mean: 21.5 years<br>SD: 20.1 years<br>Median: 16.5 years<br>Range: 2-68 years	/	0% (0/8)
Majandar, A et al. 2017 [3]	13	/	Mean: 6.8<br>SD: 3.5<br>Median: 8.0<br>Range: 2-11	Mean: 22.2 years<br>SD: 20.4 years<br>Median: 17.0 years<br>Range: 1-56 years	Improvement<br>in BCVA from baseline by two lines or more on the ETDRS chart<br>or from off-chart to on-chart visual acuity	23.08% (3/13)
Mashima, Y. et al. 2017 [4]	61<br>(40 no treatment, 21 on idebenone)	/	Mean: 23.1<br>SD: 12.1<br>Median: 18<br>Range: 9-65	3-10 years	Final VA ≥ 0.2 Snellen decimal	20.00% (8/40)
do VF Ramos, C. et al. 2009 [5]	45	/	/	≥ 1 year	/	37.78% (17/45)

Sadun, F. et al. 2004 [6]	20	Mean: 47<br>SD: 17<br>Range: 14-84	Mean: 26.11<br>SD: 9.76<br>Median: 29<br>Range: 10-41	Mean: 18.83 years<br>SD: 16.92 years<br>Median: 18 years<br>Range: 0-64 years	/	0% (0/20)
Spruijt, L. et al. 2006 [7]	82	/	/	7 patients <1 year,<br>4 patients 1-4 years,<br>7 patients >4 years	Improvement of visual acuity after onset	21.95% (18/82)
Ahn, Y. J. et al. [8]	7	/	Mean: 8.57<br>SD: 4.12<br>Median: 10<br>Range: 4-13	/	Improvement in BCVA by two lines or more on the vision chart from baseline based on the logMAR scale	57.14% (4/7)

SD = Standard Deviation; VA = Visual Acuity; BCVA = Best-corrected Visual Acuity

Reference

1. Lam BL, Feuer WJ, Abukhalil F, Porciatti V, Hauswirth WW, Guy J. Leber hereditary optic neuropathy gene therapy clinical trial recruitment: year 1. *Arch Ophthalmol* 2010; **128**: 1129-35.
2. Lu Q, Guo Y, Yi J, Deng X, Yang Z, Yuan X, Deng H. Identification of an ND4 Mutation in Leber Hereditary Optic Neuropathy. *Optom Vis Sci* 2017; **94**: 1090-4.
3. Majander A, Bowman R, Poulton J, Antcliff RJ, Reddy MA, Michaelides M, Webster AR, Chinnery PF, Votruba M, Moore AT, Yu-Wai-Man P. Childhood-onset Leber hereditary optic neuropathy. *Br J Ophthalmol* 2017; **101**: 1505-9.
4. Mashima Y, Kigasawa K, Shinoda K, Wakakura M, Oguchi Y. Visual prognosis better in eyes with less severe reduction of visual acuity one year after onset of Leber hereditary optic neuropathy caused by the 11,778 mutation. *BMC Ophthalmol* 2017; **17**: 192.
5. Ramos Cdo V, Bellusci C, Savini G, Carbonelli M, Berezovsky A, Tamaki C, Cinoto R, Sacai PY, Moraes-Filho MN, Miura HM, Valentino ML, Iommarini L, De Negri AM, Sadun F, Cortelli P, Montagna P, Salomao SR, Sadun AA, Carelli V, Barboni P. Association of optic disc size with development and prognosis of Leber's hereditary optic

neuropathy. *Invest Ophthalmol Vis Sci* 2009; **50**: 1666-74.

6. Sadun F, De Negri AM, Carelli V, Salomao SR, Berezovsky A, Andrade R, Moraes M, Passos A, Belfort R, da Rosa AB, Quiros P, Sadun AA. Ophthalmologic findings in a large pedigree of 11778/Haplogroup J Leber hereditary optic neuropathy. *Am J Ophthalmol* 2004; **137**: 271-7.
7. Spruijt L, Kolbach DN, de Coo RF, Plomp AS, Bauer NJ, Smeets HJ, de Die-Smulders CE. Influence of mutation type on clinical expression of Leber hereditary optic neuropathy. *Am J Ophthalmol* 2006; **141**: 676-82.
8. Ahn YJ, Park Y, Shin SY, Chae H, Kim M, Park SA-O. Genotypic and phenotypic characteristics of Korean children with childhood-onset Leber's hereditary optic neuropathy. *Graefes Arch Clin Exp Ophthalmol* 2020; **258**: 2283-90.
