# Supplementary material for: Leber's Hereditary Optic Neuropathy with Mitochondrial DNA Mutation G11778A: A Systematic Literature Review and Meta-Analysis
Source: Biomed Res Int. 2023 Jan 24;2023:1107866. doi: 10.1155/2023/1107866 (PMC9893526; doi:10.1155/2023/1107866)
Supplement: Supplementary 3 — S. Table 3: data summary of the previously published G11778A LHON patients with onset of visual loss less than 18 years of age. [file 1107866.f3.pdf]

Table S3. Data summary of previously published G11778A LHON patients with onset of visual loss less than 18 years of age.

Author	Patients	Sex		Age at onset (yrs)	BCVA (LogMAR)*		
	n	Female	Male	Mean (SD)<br>Median (IQR)	Mean (SD)<br>Median (IQR)	> 1·30<br>(%)	≤ 0·30<br>(%)
Jiang, P et al. 2015 [1]	68	9	59	14.3 (2.92)<br>15.0 (3.00)	1.35 (0.53)<br>1.30 (0.52)	73/136<br>(53.7)	1/136<br>(0.7)
Lu, Q et al. 2017 [2]	5	2	3	12.6 (2.30)<br>13.0 (2.00)	1.02 (0.74)<br>0.849 (0.43)	2/10<br>(20.0)	1/10<br>(10.0)
Majandar, A et al. 2017 [3]	13	3	10	6.85 (3.46)<br>8.00 (6.00)	1.22 (0.91)<br>1.00 (1.21)	9/26<br>(34.6)	2/26<br>(7.7)
Qiao,C et al. 2015 [4]	3	3	0	15.3 (2.89)<br>17.0 (2.50)	1.83 (0.53)<br>1.85 (0.60)	6/6<br>(100.0)	0/6<br>(0.0)
Sadun, F et al. 2004 [5]	5	0	5	13.6 (2.88)<br>13.0 (1.00)	1.71 (0.47)<br>1.69 (0.53)	7/10<br>(70.0)	3/10<br>(30.0)
Wan, X et al. 2016 [6]	8	2	6	11.0 (3.38)<br>11.0 (5.25)	1.65 (0.44)<br>1.70 (0.80)	5/8<br>(62.5)	0/8<br>(0.0)
Ahn, Y. J. et al.2020 [7]	12	2	10	8.0 (3.93)<br>7.5 (6.75)	0.60 (0.73)<br>0.35 (0.90)	5/24<br>(20.8)	11/24<br>(45.8)

SD = Standard Deviation; IQR = Inter-quartile Range; BCVA = Best-corrected Visual Acuity

* Number of eyes with best-corrected visual acuity (BCVA) > 1.3 or ≤ 0.3 in logarithm of minimum angle of resolution (LogMAR).

Reference

1. Jiang P, Liang M, Zhang J, Gao Y, He Z, Yu H, Zhao F, Ji Y, Liu X, Zhang M, Fu Q, Tong Y, Sun Y, Zhou X, Huang T, Qu J, Guan MX. Prevalence of Mitochondrial ND4 Mutations in 1281 Han Chinese Subjects With Leber's Hereditary Optic Neuropathy. *Invest Ophthalmol Vis Sci* 2015; **56**: 4778-88.
2. Lu Q, Guo Y, Yi J, Deng X, Yang Z, Yuan X, Deng H. Identification of an ND4 Mutation in Leber Hereditary Optic Neuropathy. *Optom Vis Sci* 2017; **94**: 1090-4.
3. Majander A, Bowman R, Poulton J, Antcliff RJ, Reddy MA, Michaelides M, Webster AR, Chinnery PF, Votruba M, Moore AT, Yu-Wai-Man P. Childhood-onset Leber hereditary optic neuropathy. *Br J Ophthalmol* 2017; **101**: 1505-9.
4. Qiao C, Wei T, Hu B, Peng C, Qiu X, Wei L, Yan M. Two families with Leber's hereditary optic neuropathy carrying G11778A and T14502C mutations with haplogroup H2a2a1 in mitochondrial DNA. *Mol Med Rep* 2015; **12**: 3067-72.
5. Sadun F, De Negri AM, Carelli V, Salomao SR, Berezovsky A, Andrade R, Moraes M, Passos A, Belfort R, da Rosa AB, Quiros P, Sadun AA. Ophthalmologic findings in a large pedigree of 11778/Haplogroup J Leber hereditary optic neuropathy. *Am J Ophthalmol* 2004; **137**: 271-7.
6. Wan X, Pei H, Zhao MJ, Yang S, Hu WK, He H, Ma SQ, Zhang G, Dong XY, Chen C, Wang DW, Li B. Efficacy and Safety of rAAV2-ND4 Treatment for Leber's Hereditary Optic Neuropathy. *Sci Rep* 2016; **6**: 21587.
7. Ahn YJ, Park Y, Shin SY, Chae H, Kim M, Park SA-O. Genotypic and phenotypic characteristics of Korean children with childhood-onset Leber's hereditary optic neuropathy. *Graefes Arch Clin Exp Ophthalmol* 2020; **258**: 2283-90.
