# Supplementary material for: Leber's Hereditary Optic Neuropathy with Mitochondrial DNA Mutation G11778A: A Systematic Literature Review and Meta-Analysis
Source: Biomed Res Int. 2023 Jan 24;2023:1107866. doi: 10.1155/2023/1107866 (PMC9893526; doi:10.1155/2023/1107866)
Supplement: Supplementary 4 — S. Figure 1: systematic literature review protocol. [file 1107866.f4.pdf]

Suplementary 1. Systematic Literature Review Protocol

OBJECTIVE

- To conduct systematic literature review on the epidemiology (incidence, prevalence, age, gender, ethnicity, etc.), diagnosis, treatment, prognosis (visual acuity loss, recovery, etc.) and quality of life of LHON with *G11778A* mutation.
- To conduct meta-analysis on outcome measures of interest such as prevalence, visual acuity loss, vision recovery, disease onset age, gender ratio for LHON with *G11778A* mutation, if feasible.

REVIEW QUESTION

To inform the review objective, the population, intervention, comparison, outcome and study type (PICOS) framework is shown in Table 1.

Table 1. PICOS framework to inform review objective.

Component	PICOS Details
Population	• Patients diagnosed with Leber Hereditary Optic Neuropathy with G11778A mutation
Intervention	• No restriction
Comparison	• No restriction
Outcome	• Epidemiology, diagnosis/testing, treatment, prognosis, quality of life, clinical guideline and consensus
Study type	• No restriction

LITERATURE SEARCH

The Medline (PubMed) database will be used to perform the literature search. Currently, the review data source in scope only includes PubMed as the only literature database.

The search strategy comprises of two main components for this review, population and outcome. Both keywords (free text words) and index terms (subject headings) will be used to create a comprehensive search strategy. The exact search terms for PubMed are shown in Table 2.

All search result citations will be imported into and managed by the EndNote bibliographic software. Duplicate citations will be removed prior to downstream article screening.

Table 2. Search string prepared for PubMed search engine

	Search Terms

Population	("Optic Atrophy, Hereditary, Leber") AND (11778* OR m.11778* OR G11778* OR ND4/11778*)	
Outcome	a) Epidemiology:	Incidence [Title/Abstract] OR Prevalence [Title/Abstract] OR Gender Bias [Title/Abstract] OR "Sexism" [Mesh]
	b) Diagnosis:	diagnosis [Title/Abstract] OR testing [Title/Abstract]
	c) Treatment:	Estrogen [Title/Abstract] OR "idebenone" [Supplementary Concept] OR "Alpha-tocotrienol Quinone"[Supplementary Concept] OR "Hormone Replacement Therapy"[Mesh] OR "Estrogen Replacement Therapy"[Mesh] OR "Mitochondrial Replacement Therapy"[Mesh] OR "Genetic Therapy"[Mesh]
	d) Prognosis:	Visual Acuity/Drug Effects [Title/Abstract] OR Recovery [Title/Abstract]
	e) Quality of life:	"Quality of Life"[Mesh]
	f) Clinical guideline/consensus:	"Guidelines as Topic"[Mesh] OR "Consensus"[Mesh]

STUDY SELECTION

Study selection through screening process will be piloted and tested by two reviewers based on a subset of citations (5% of citations if n > 50 or 10% of citations if n ≤ 50, while n is the total number of citations across the search engine with the removal of duplicates). The actual screening process involves two stages of independent screening of literature articles.

In the first stage, two reviewers with training in systematic literature review will independently conduct screening at title and abstract level and identify articles for full article screening later. In Table 3, each evaluation question will be answered as "Yes", "No", or "Unclear". The articles will be excluded if both reviewers answer "No" to any of the four questions. If both reviewers answer "Yes" and/or "Unsure" to all questions, the article will be included for full article screening later. Any disagreements will be resolved by consensus. When consensus cannot be reached, a third reviewer will arbitrate.

In the second stage, full article screening will be conducted on the remaining articles after the title and abstract screening. Similarly, two reviewers will screen articles independently based on the question in Table 4, in addition to all the domains in Table 3 at full article level. In full article screening, reviewers will identify all articles using the duplicate dataset and will keep the article that is the most comprehensive and recent. Each question in Table 4 will be answered as either "Yes" or "No". The articles will be excluded if both reviewers answered "No" to any question. Any disagreement will be resolved by discussion between the paired reviewers to reach consensus. If consensus cannot be reached, a third reviewer will independently appraise the article and discuss with the other two reviewers to reach consensus.

Table 3. Evaluation questions for title and abstract screening.

Domain	Question	Evaluation Guideline		
		Yes	No	Unclear
Research Design	Do the title and abstract of the article describe an epidemiological study?	Include	Exclude	Include
Publication Source	Do the title and abstract of the article come from a published study?	Include	Exclude	Include
Publication Type	Is the published study not a non-target mutation (nonG11778A) study, nonhuman study, basic research study (e.g. genetics), traditional Chinese medicine study, review study, case report, or thesis?	Include	Exclude	Include
Population	Does the population of interest include patients with Leber hereditary optic neuropathy and with G11778A mutation?	Include	Exclude	Include

Table 4. Additional evaluation question for full article screening.

Domain	Question	Evaluation Guideline	
		Yes	No
Duplicate	Does the research use new dataset for analysis?<br>(i.e. dataset is not previously analyzed in another included study)	Include	Exclude<br>(Note: When identifying the study to be included, the most comprehensive and recent study will be used.)

DATA EXTRACTION

The following study attributes from four domains will be extracted and tabulated in Excel (Table 5). Any additional study attributes encountered during the review will be included when they are determined as relevant and appropriate information.

Table 5. Study attributes for extraction.

Data Extraction Domain	Attributes
Article Identifiers	• study title<br>• publication<br>• year journal<br>• volume<br>• issue<br>• page numbers<br>• digital object identifier
Attributes about Study	• publication type<br>• study design<br>• study location or country
Attributes about Population	• population definition<br>• population size<br>• demographic information (e.g. age, sex)
Attributes about Methods<br>(If available)	• experimental/exposure group control<br>• group
Attributes about Outcomes<br>(If available)	• onset age gender<br>• ratio/bias<br>• ethnicity<br>• incidence rate<br>• prevalence rate<br>• disease natural history (e.g. time to different levels of vision loss) diagnosis or testing treatment or intervention<br>• adverse effects of treatment or intervention<br>• family history<br>• visual acuity<br>• contrast sensitivity

	• visual field information (e.g. visual field index)<br>• health economics related information (e.g. direct/indirect cost, productivity loss, subsidy policies)

QUALITY APPRAISAL

Assessment of the methodological quality of the literature article will be undertaken independently by two reviewers. Appraisal will be assessed The risk of bias and quality of studies were assessed by two reviewers based on Joanna Briggs Institute model of evidence-based healthcare bias assessment.[1] Disputes between reviewers were resolved through discussion until consensus was reached. For each source of bias outlined in Table 6, reviewers will assign “high risk,” “low risk,” “unclear” or “not applicable” and provide the support for judgement in brief descriptions.

Table 6. Evaluation for risk of bias[1]

Source of Bias in randomized controlled trials	Reviewer Judgement
Random sequence generation	A true random assignment of participants to the groups means that a procedure is used that allocates the participants to groups purely based on chance, not influenced by the known characteristics of the participants.
Allocation concealment	Concealment of allocation (allocation concealment) refers to procedures that prevent those allocating patients from knowing before allocation which treatment or control is next in the allocation process.
Blinding-experiment	Blinding of the participants refers to procedures that prevent participants from knowing which group they are allocated. Blinding of those delivering treatment refers to procedures that prevent those delivering treatment from knowing which group they are treating, that is those delivering treatment are not aware if they are treating the group receiving the treatment of interest or if they are treating any other group receiving the control interventions.
Blinding-observation	Blinding of outcomes assessors is used in order to minimize this risk that those assessing the outcomes are aware of participants’ allocation to the treatment group or to the control group

Incomplete outcome data	If incomplete outcome reporting is found for a given study, it suggests that the researchers cherry-picked their results and did not report all the outcomes in which they were interested when they began the study.
Selective reporting	Selective outcome reporting has been defined as the selection of a subset of the original variables recorded, on the basis of the results, for inclusion in publication of trials

**Source of Bias in nonrandomized trials**	**Reviewer Judgement**
Clear cause and effect definition	Ambiguity with regards to the temporal relationship of variables constitutes a threat to the internal validity of a study exploring causal relationships. The treatment or intervention of interest should occur in time before the explored the effect or outcome of interest.
Similarity of patients in comparison	If there are differences between participants included in compared groups there is a risk of selection bias. If there are differences between participants included in the compared groups maybe the effect cannot be attributed to the potential cause, as maybe it is plausible that the effect may be explained by the differences between participants, that is, by selection bias.
Control group exists	The validity of causal inferences is strengthened in studies with at least one independent control group compared to studies without an independent control group. Check if there are independent, separate groups, used as control groups in the study.
Multiple measurements of outcomes in pre/post intervention	Check if measurements were collected before the intervention of interest was implemented. If there is no measurement before the treatment and only measurement after the treatment is available, it is not known if there is a change after the treatment compared to before the treatment.
Sufficient follow-up	Check if there were differences with regards to the loss to follow up between the compared groups. If follow up was incomplete, examine the reported details about the strategies used in order to address incomplete follow up, such as descriptions of loss to follow up and impact analyses.

Consistent outcome measurement	Check if the outcomes were measured in the same way. If the outcome is not measured in the same way in the compared groups there is a threat to the internal validity of a study exploring a causal relationship as the differences in outcome measurements may be confused with an effect of the treatment or intervention of interest.
Outcome measured in valid and reliable way	Check the details about the reliability of measurement such as the number of raters, training of raters, the intra-rater reliability, and the inter-raters reliability within the study. Unreliability of outcome measurements is one threat that weakens the validity of inferences about the statistical relationship between the ‘cause’ and the ‘effect’ estimated in a study exploring causal effects.

Appropriate statistics used	Check the following aspects: if the assumptions of statistical tests were respected; if appropriate statistical power analysis was performed; if appropriate effect sizes were used; if appropriate statistical procedures or methods were used given the number and type of dependent and independent variables, the number of study groups, the nature of the relationship between the groups , and the objectives of statistical analysis.
**Source of Bias in crosssectional studies**	**Reviewer Judgement**
Clear inclusion criteria	The authors should provide clear inclusion and exclusion criteria that they developed prior to recruitment of the study participants. The inclusion/exclusion criteria should be specified (e.g., risk, stage of disease progression) with sufficient detail and all the necessary information critical to the study.
Detailed description of subjects and setting	The study sample should be described in sufficient detail so that other researchers can determine if it is comparable to the population of interest to them. The authors should provide a clear description of the population from which the study participants were selected or recruited, including demographics, location, and time period.
Valid exposure measurement	The study should clearly describe the method of measurement of exposure. Assessing validity requires that a 'gold standard' is available to which the measure can be compared. The validity of exposure measurement usually relates to whether a current measure is appropriate or whether a measure of past exposure is needed.

Confounding addressed	A confounder is a difference between the comparison groups, and it influences the direction of the study results. A high-quality study at the level of cohort design will identify the potential confounders and measure them (where possible). Look out for a description of statistical methods as regression methods such as logistic regression are usually employed to deal with confounding factors/ variables of interest.
Valid outcome measurement	Determine if the measurement tools used were validated instruments as this has a significant impact on outcome assessment validity. Having established the objectivity of the outcome measurement instrument, it's important to establish how the measurement was conducted.
Appropriate statistical analysis	As with any consideration of statistical analysis, consideration should be given to whether there was a more appropriate alternate statistical method that could have been used. The methods section should be detailed enough for reviewers to identify which analytical techniques were used (regression or stratification) and how specific confounders were measured.
**Source of Bias in casecontrol studies**	**Reviewer Judgement**
Groups comparability other than the presence of disease in cases or the absence of disease in controls	The control group should be representative of the source population that produced the cases. wherein controls are selected for each case based on similarity with respect to certain characteristics other than the exposure of interest. Selection bias may result if the groups are not comparable.
Appropriate match between case and control	The study should include clear definitions of the source population. Sources from which cases and controls were recruited should be carefully looked at. Study participants may be selected from the target population, the source population, or from a pool of eligible participants (such as in hospital-based case control studies).
Valid and reliable exposure measurement	The study should clearly describe the method of measurement of exposure. Assessing validity requires that a 'gold standard' is available to which the measure can be compared. The validity of exposure measurement usually relates to whether a current measure is appropriate or whether a measure of past exposure is needed.

Confounding addressed	A confounder is a difference between the comparison groups, and it influences the direction of the study results. A high-quality study at the level of case control design will identify the potential confounders and measure them (where possible). Look out for a description of statistical methods as regression methods such as logistic regression are usually employed to deal with confounding factors/ variables of interest.
Consistent outcome assessment between case and control	Determine if the measurement tools used were validated instruments as this has a significant impact on outcome assessment validity. Having established the objectivity of the outcome measurement instrument, it's important to establish how the measurement was conducted.
Sufficient exposure duration	It is particularly important in a case control study that the exposure time was enough to show an association between the exposure and the outcome. It may be that the exposure period may be too short or too long to influence the outcome

Appropriate statistical analysis	The methods section should be detailed enough for reviewers to identify which analytical techniques were used (in particular, regression or stratification) and how specific confounders were measured.
**Source of Bias in cohort studies**	**Reviewer Judgement**
Group similarity	Check the paper carefully for descriptions of participants to determine if patients within and across groups have similar characteristics in relation to exposure. The two groups selected for comparison should be as similar as possible in all characteristics except for their exposure status, relevant to the study in question.
Consistent, valid exposure measurement for exposed/unexposed group	The exposure measures should be clearly defined and described in detail. This will enable reviewers to assess whether the participants received the exposure of interest. Assessing validity requires that a 'gold standard' is available to which the measure can be compared. The validity of exposure measurement usually relates to whether a current measure is appropriate or whether a measure of past exposure is needed.

Confounding addressed	A confounder is a difference between the comparison groups, and it influences the direction of the study results. A high-quality study at the level of cohort design will identify the potential confounders and measure them (where possible). Look out for a description of statistical methods as regression methods such as logistic regression are usually employed to deal with confounding factors/ variables of interest.
Groups are free of outcome at the start of study	The participants should be free of the outcomes of interest at the start of the study. Refer to the 'methods' section in the paper for this information, which is usually found in descriptions of participant/sample recruitment, definitions of variables, and/or inclusion/exclusion criteria.
Valid outcome measure	Determine if the measurement tools used were validated instruments as this has a significant impact on outcome assessment validity. Having established the objectivity of the outcome measurement instrument, it's important to establish how the measurement was conducted.
Sufficient follow-up time	The appropriate length of time for follow up will vary with the nature and characteristics of the population of interest and/or the intervention, disease or exposure. To estimate an appropriate duration of follow up, read across multiple papers and take note of the range for duration of follow up.

Follow-up completion	It is important in a cohort study that a greater percentage of people are followed up. As a general guideline, at least 80% of patients should be followed up. Generally, a dropout rate of 5% or less is considered insignificant. A rate of 20% or greater is considered to significantly impact on the validity of the study. Reporting of efforts to follow up participants that dropped out may be regarded as an indicator of a well conducted study.
Incomplete follow-up addressed	Selection bias may occur as a result of incomplete follow up. Therefore, participants with unequal follow up periods must be considered in the analysis, which should be adjusted to allow for differences in length of follow up periods. This is usually done by calculating rates which use personyears at risk, i.e. considering time in the denominator.
Appropriate statistical analysis	The methods section should be detailed enough for reviewers to identify which analytical techniques were used (regression or stratification) and how specific confounders were measured.
**Source of Bias in case series studies**	**Reviewer Judgement**

Clear inclusion criteria	The authors should provide clear inclusion (and exclusion criteria where appropriate) for the study participants. The inclusion/exclusion criteria should be specified (e.g., risk, stage of disease progression) with sufficient detail and all the necessary information critical to the study.
Condition measured in a standard and reliable way	The study should clearly describe the method of measurement of the condition. This should be done in a standard (i.e. same way for all patients) and reliable (i.e. repeatable and reproducible results) way.
Valid method for patient identification	Many health problems are not easily diagnosed or defined, and some measures may not be capable of including or excluding appropriate levels or stages of the health problem. If the outcomes were assessed based on existing definitions or diagnostic criteria, then the answer to this question is likely to be yes. If the outcomes were assessed using observer reported, or self-reported scales, the risk of over- or under-reporting is increased, and objectivity is compromised.
Consecutive participants inclusion	Studies that indicate a consecutive inclusion are more reliable than those that do not. For example, a case series that states, 'we included all patients with osteosarcoma who presented to our clinic between March 2005 and June 2006' is more reliable than a study that simply states 'we report a case series of 24 people with osteosarcoma.'
Complete inclusion of patients	The completeness of a case series contributes to its reliability. Studies that indicate a complete inclusion are more reliable than those that do not.
Clear demographic information reported	The case series should clearly describe relevant participant's demographics such as the following information where relevant: participant's age, sex, education, geographic region, ethnicity, time period, education.
Clear clinical information reported	There should be clear reporting of clinical information of the participants such as the following information where relevant: disease status, comorbidities, stage of disease, previous interventions/treatment, results of diagnostic tests, etc.
Clear outcomes or follow-up results reporting	The results of any intervention or treatment should be clearly reported in the case series. A good case study should clearly describe the clinical condition post-intervention in terms of the presence or lack of symptoms.

Clear presenting site(s)/clinic(s) demographic information reporting	Certain diseases or conditions vary in prevalence across different geographic region and population (e.g. women vs. men, sociodemographic variable between countries). The study sample should be described in sufficient detail so that other researchers can determine if it is comparable to the population of interest to them
Appropriate statistics used	The methods section of studies should be detailed enough for reviewers to identify which analytical techniques were used and whether these were suitable.

SYNTHESIS REPORT

Based on compiling evidence from the review, current research and existing evidence gaps related to the epidemiology, diagnosis, treatment, prognosis and quality of life will be highlighted in a synthesis report. The report will document the review findings following the PRISMA Checklist. The report will contain 27 items detailed in Appendix A. Appropriate figures and/or tables will be included in the report for visualization and/or tabulation.

REFERENCES

1. Institute, J.B., *Joanna Briggs Institute reviewers' manual: 2014 edition.* Australia: The Joanna Briggs Institute, 2014.

APPENDIX A

PRISMA checklist for synthesis report

Section/Topic	Number	Item
TITLE		
Title	1	Identify the report as a systematic review, meta-analysis, or both.
ABSTRACT		
Structured summary	2	Provide a structured summary including, as applicable: background; objectives; data sources; study eligibility criteria, participants, and interventions; study appraisal and synthesis methods; results; limitations; conclusions and implications of key findings; systematic review registration number.
INTRODUCTION		
Rationale	3	Describe the rationale for the review in the context of what is already known.
Objectives	4	Provide an explicit statement of questions being addressed with reference to participants, interventions, comparisons, outcomes, and study design (PICOS).
METHODS		
Protocol and registration	5	Indicate if a review protocol exists, if and where it can be accessed (e.g., Web address), and, if available, provide registration information including registration number.
Eligibility criteria	6	Specify study characteristics (e.g., PICOS, length of follow-up) and report characteristics (e.g., years considered, language, publication status) used as criteria for eligibility, giving rationale.
Information sources	7	Describe all information sources (e.g., databases with dates of coverage, contact with study authors to identify additional studies) in the search and date last searched.

Search	8	Present full electronic search strategy for at least one database, including any limits used, such that it could be repeated.
Study selection	9	State the process for selecting studies (i.e., screening, eligibility, included in systematic review, and, if applicable, included in the meta-analysis).
Data collection process	10	Describe method of data extraction from reports (e.g., piloted forms, independently, in duplicate) and any processes for obtaining and confirming data from investigators.
Data items	11	List and define all variables for which data were sought (e.g., PICOS, funding sources) and any assumptions and simplifications made.
Risk of bias in individual studies	12	Describe methods used for assessing risk of bias of individual studies (including specification of whether this was done at the study or outcome level), and how this information is to be used in any data synthesis.
Summary measures	13	State the principal summary measures (e.g., risk ratio, difference in means).
Synthesis of results	14	Describe the methods of handling data and combining results of studies, if done, including measures of consistency for each meta-analysis.
Risk of bias across studies	15	Specify any assessment of risk of bias that may affect the cumulative evidence (e.g., publication bias, selective reporting within studies).
Additional analyses	16	Describe methods of additional analyses (e.g., sensitivity or subgroup analyses, meta-regression), if done, indicating which were pre-specified.
RESULTS		
Study selection	17	Give numbers of studies screened, assessed for eligibility, and included in the review, with reasons for exclusions at each stage, ideally with a flow diagram.
Study characteristics	18	For each study, present characteristics for which data were extracted (e.g., study size, PICOS, follow-up period) and provide the citations.

Risk of bias within studies	19	Present data on risk of bias of each study and, if available, any outcome level assessment (see item 12).
Results of individual studies	20	For all outcomes considered (benefits or harms), present, for each study: (a) simple summary data for each intervention group (b) effect estimates and confidence intervals, ideally with a forest plot.
Synthesis of results	21	Present results of each meta-analysis done, including confidence intervals and measures of consistency.
Risk of bias across studies	22	Present results of any assessment of risk of bias across studies (see Item 15).
Additional analysis	23	Give results of additional analyses, if done (e.g., sensitivity or subgroup analyses, meta-regression [see Item 16]).
DISCUSSION		
Summary of evidence	24	Summarize the main findings including the strength of evidence for each main outcome; consider their relevance to key groups (e.g., healthcare providers, users, and policy makers).
Limitations	25	Discuss limitations at study and outcome level (e.g., risk of bias), and at review-level (e.g., incomplete retrieval of identified research, reporting bias).
Conclusions	26	Provide a general interpretation of the results in the context of other evidence, and implications for future research.
FUNDING		
Funding	27	Describe sources of funding for the systematic review and other support (e.g., supply of data); role of funders for the systematic review.
