# Supplementary material for: Leber's Hereditary Optic Neuropathy with Mitochondrial DNA Mutation G11778A: A Systematic Literature Review and Meta-Analysis
Source: Biomed Res Int. 2023 Jan 24;2023:1107866. doi: 10.1155/2023/1107866 (PMC9893526; doi:10.1155/2023/1107866)
Supplement: Supplementary 5 — S. Figure 2: evaluation questions for title and abstract screening. [file 1107866.f5.pdf]

Suplementary 2. Evaluation questions for title and abstract screening.

**Domain**	**Question**	**Evaluation Guideline**		
		**Yes**	**No**	**Unclear**
Research Design	Do the title and abstract of the article describe an epidemiological study?	Include	Exclude	Include
Publication Source	Do the title and abstract of the article come from a published study?	Include	Exclude	Include
Publication Type	Is the published study not a non-target mutation (non-G11778A) study, non-human study, basic research study (e.g. genetics), traditional Chinese medicine study, review study, case report, or thesis?	Include	Exclude	Include
Population	Does the population of interest include patients with Leber hereditary optic neuropathy and with G11778A mutation?	Include	Exclude	Include
