# Supplementary material for: Leber's Hereditary Optic Neuropathy with Mitochondrial DNA Mutation G11778A: A Systematic Literature Review and Meta-Analysis
Source: Biomed Res Int. 2023 Jan 24;2023:1107866. doi: 10.1155/2023/1107866 (PMC9893526; doi:10.1155/2023/1107866)
Supplement: Supplementary 6 — S. Figure 3: risk of bias assessment. [file 1107866.f6.pdf]

Suplementary 3:

a) Risk of bias table – case series

	Clear inclusion criteria	Condition measured in a standard, reliable way	Valid method for patient identification	Consecutive participants inclusion	Complete inclusion of patients	Clear demographic information reporting	Clear clinical information reporting	Clear outcomes or follow-up results reporting	Clear presenting site(s)/clinic(s) demographic information reporting	Appropriate statistics used
Chuenkongkaew, W. L. 2005	High risk	Low risk	Unclear	Unclear	High risk	High risk	Low risk	Unclear	High risk	Low risk
Cui, G. 2013	Unclear	Low risk	Low risk	Low risk	Unclear	High risk	Unclear	Low risk	Low risk	Low risk
Dimitriadis, K. 2014	High risk	Low risk	Unclear	Unclear	Unclear	Low risk	Low risk	Low risk	Unclear	Low risk
Du, W. D. 2011	Low risk	Low risk	Low risk	Low risk	Unclear	High risk	Low risk	Low risk	Low risk	Not applicable
Guy, J. 2014	High risk	Low risk	High risk	High risk	Unclear	Low risk	Low risk	Low risk	Unclear	Low risk
Ji, Y. 2008	High risk	Low risk	Unclear	Unclear	Unclear	High risk	Low risk	Low risk	High risk	Not applicable
Jiang, P. 2015	High risk	Low risk	Unclear	Unclear	Low risk	Low risk	Low risk	Low risk	High risk	Low risk
Khan, N. A. 2017	Low risk	Low risk	Low risk	Low risk	Unclear	Low risk	Low risk	Low risk	Low risk	Low risk
Kim, J. Y. 2003	Low risk	Low risk	Low risk	Unclear	Unclear	High risk	Low risk	Low risk	Unclear	Unclear
Kirkman, M. A. 2009	High risk	Low risk	Low risk	Unclear	Unclear	Low risk	Low risk	Low risk	Low risk	Low risk
Lam, B. L. 2014	High risk	Low risk	High risk	Low risk	Unclear	Low risk	Low risk	Low risk	High risk	Low risk
Lu, Q. 2017	Low risk	Low risk	Low risk	High risk	High risk	Low risk	Low risk	Low risk	Low risk	Not applicable
Majander, A. 2017	Low risk	Low risk	Low risk	Unclear	Low risk	Low risk	Low risk	Low risk	Low risk	Low risk
Mashima, Y. 2017	High risk	Low risk	Low risk	Low risk	Unclear	Low risk	Low risk	Low risk	Low risk	Low risk
Mishra, A. 2017	High risk	Low risk	Low risk	Low risk	Low risk	Low risk	Low risk	Low risk	Low risk	Not applicable
Qiao, C. 2015	High risk	Low risk	High risk	High risk	High risk	Low risk	Low risk	Low risk	High risk	High risk
Ramos Cdo, V. 2009	Low risk	Low risk	Low risk	Low risk	Low risk	Low risk	Low risk	Low risk	Low risk	Low risk
Sadun, A. A. 2002	Low risk	Low risk	Low risk	Low risk	Low risk	Low risk	Low risk	Low risk	Low risk	Low risk
Sadun, A. A. 2003	Low risk	Low risk	Low risk	Low risk	Low risk	Low risk	Low risk	Low risk	Low risk	Low risk
Sadun, F. 2004	Low risk	Low risk	Low risk	Low risk	Low risk	Low risk	Low risk	Low risk	Low risk	Unclear
Sadun, A. A. 2006	Low risk	Low risk	Low risk	Low risk	Low risk	Low risk	Low risk	Low risk	Low risk	Unclear
Spruijt, L. 2006	Unclear	Unclear	Low risk	High risk	High risk	Low risk	Low risk	Low risk	Low risk	Low risk
Marotta, R. 2020	Low risk	Low risk	High risk	Low risk	Low risk	Low risk	Low risk	Low risk	Low risk	Low risk
Tonagel, F. 2021	Low risk	Unclear	Unclear	Low risk	Low risk	Low risk	Low risk	Low risk	Low risk	Unclear
Ahn, Y. J. 2020	Low risk	Low risk	Low risk	Unclear	Unclear	Low risk	Low risk	Low risk	Low risk	Low risk
Poincenot, L. 2020	Low risk	High risk	High risk	Unclear	Unclear	Low risk	Low risk	Low risk	Low risk	Low risk
Cui, S. 2019	Low risk	Low risk	Low risk	High risk	High risk	Low risk	Low risk	Low risk	Low risk	Low risk
Dokrungkoon, T. 2019	Low risk	Low risk	Low risk	Unclear	Unclear	High risk	Low risk	Low risk	Low risk	Low risk
Liu, H. L. 2019	Low risk	Low risk	Unclear	Unclear	Unclear	Low risk	Low risk	Low risk	High risk	Low risk
Zhao, X. 2020	Low risk	Low risk	Low risk	Low risk	Low risk	High risk	Low risk	Low risk	Low risk	Low risk
Li, J. K. 2020	Low risk	Low risk	Low risk	Unclear	Unclear	Low risk	Low risk	Low risk	Low risk	Low risk

(green) = Low risk (red) = High risk (yellow) = Unclear (white) = Not applicable

b) Risk of bias table – Studies reporting prevalence data

	Appropriate sample frame	Appropriate sampling method	Adequate sample size	Detailed description on subjects and setting	Data analysis with sufficient coverage on identified sample	Standard, reliable condition measurement on participants	Appropriate statistical analysis	Adequate response rate
Ueda, K. 2017	Low risk	Low risk	Low risk	High risk	Unclear	Low risk	Low risk	Low risk
Gowri, P. 2020	Low risk	Low risk	Low risk	Low risk	Low risk	Low risk	Low risk	Low risk

= Lowrisk = Highrisk =Unclear = Not applicable

c) Risk of bias table – non-randomized clinical trials

	Clear cause and effect	Similarity of patients in comparisons	Control group exists	Multiple measurements of outcomes in pre/post intervention	Sufficient follow-up	Consistent outcome measurement	Outcome measured in valid and reliable way	Appropriate statistics used
Feuer, W. J. 2016	Low risk	Low risk	Low risk	Low risk	Low risk	Low risk	Low risk	Low risk
Guy, J. 2017	Low risk	Low risk	Low risk	Low risk	Low risk	Low risk	Low risk	Low risk
Wan, X. 2016	Low risk	Not applicable	High risk	Low risk	Low risk	Low risk	Low risk	Low risk
Ishikawa, H. 2021	Low risk	Not applicable	High risk	Low risk	Low risk	Low risk	Low risk	Low risk
Yang, S. 2020	Low risk	Not applicable	High risk	Low risk	Low risk	Low risk	Low risk	Low risk

= Low risk = High risk = Unclear = Not applicable

d) Risk of bias table – Randomized-control trial

	Random sequence generation	Allocation concealment	Blinding- experimentation	Blinding- observation	Incomplete outcome data	Selective reporting
Klopstock, T. 2011	Low risk	Low risk	Low risk	Unclear	Low risk	Low risk
Yu-Wai-Man, P. 2020	Low risk	Low risk	High risk	Low risk	Low risk	Low risk
Newman, N.J. 2021	Low risk	Low risk	High risk	Low risk	Low risk	Low risk

= Low risk = High risk = Unclear = Not applicable
