# Supplementary material for: Leber's Hereditary Optic Neuropathy with Mitochondrial DNA Mutation G11778A: A Systematic Literature Review and Meta-Analysis
Source: Biomed Res Int. 2023 Jan 24;2023:1107866. doi: 10.1155/2023/1107866 (PMC9893526; doi:10.1155/2023/1107866)

Study	Cases	Total	Mutation Rate	95% CI
Dimitriadis, K. 2014	167	253	0.66	[0.60; 0.72]
Du, W. D. 2011	10	12	0.83	[0.52; 0.96]
Kim, J. Y. 2003	46	60	0.77	[0.64; 0.86]
Kirkman, M. A. 2009	132	196	0.67	[0.60; 0.74]
Klopstock, T. 2011	57	85	0.67	[0.56; 0.76]
Majander, A. 2017	13	27	0.48	[0.30; 0.66]
Ramos Cdo, V. 2009	45	66	0.68	[0.56; 0.78]
Spruijt, L. 2006	145	325	0.45	[0.39; 0.50]
Ueda, K. 2017	38	44	0.86	[0.73; 0.94]
Tonagel, F. 2021	18	22	0.82	[0.60; 0.93]
Ishikawa, H. 2021	54	57	0.95	[0.85; 0.98]
Marotta, R. 2020	52	76	0.68	[0.57; 0.78]
Li, J. K. 2020	84	96	0.88	[0.79; 0.93]
Ahn, Y. J. 2020	38	44	0.86	[0.73; 0.94]
Poincenot, L. 2020	1044	1497	0.70	[0.67; 0.72]
Zhao, X. 2020	29	33	0.88	[0.72; 0.95]
Dokrungkoon, T. 2019	10	13	0.77	[0.48; 0.92]
**Random effects model**		**.**	**0.75**	**[0.67; 0.81]**

Heterogeneity: $I^2 = 88\%$, $\tau^2 = 0.4849$, $\chi^2_{16} = 133.20$ ($p < 0.01$)

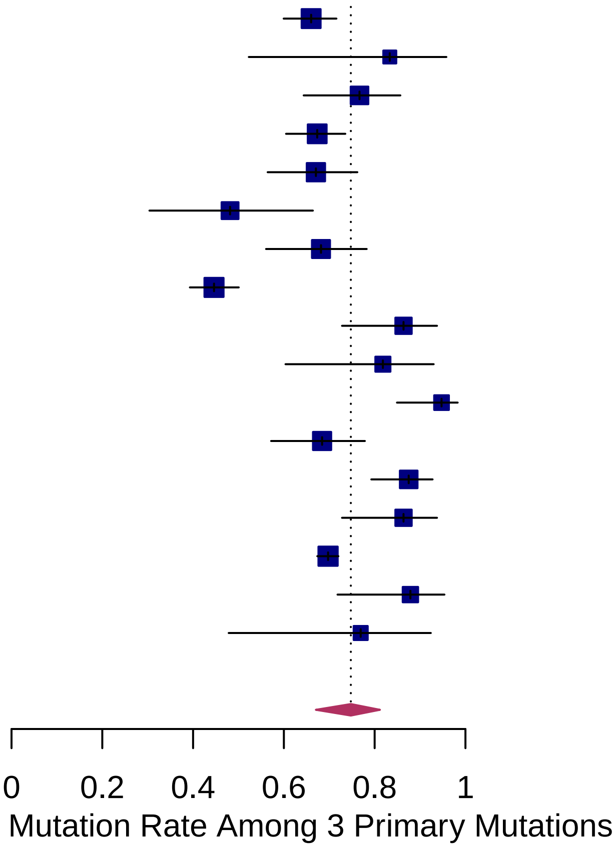

Supplement: Supplementary 7 — S. Figure 4-A: forest plot of G11778A mutation rate among 3 primary LHON mutations. S. Figure 4-B: leave-one-out analysis of studies reporting G11778A mutation rate among 3 primary LHON mutations. S. Figure 4-C: potential outliers identified from K-means clustering, DBSCAN, and Gaussian mixture models in studies reporting G11778A mutation rate among 3 primary LHON mutations. S. Figure 4-D: the Baujat plot of the influence of remaining studies reporting G11778A mutation rate among 3 primary LHON mutations after excluding potential outliers identified previously by K-means clustering, DBSCAN, and Gaussian mixture models. [file 1107866.f7.zip › S. Figure 4-A_SuppInfo.pdf]
