# Supplementary material for: Leber's Hereditary Optic Neuropathy with Mitochondrial DNA Mutation G11778A: A Systematic Literature Review and Meta-Analysis
Source: Biomed Res Int. 2023 Jan 24;2023:1107866. doi: 10.1155/2023/1107866 (PMC9893526; doi:10.1155/2023/1107866)
Supplement: Supplementary 7 — S. Figure 4-A: forest plot of G11778A mutation rate among 3 primary LHON mutations. S. Figure 4-B: leave-one-out analysis of studies reporting G11778A mutation rate among 3 primary LHON mutations. S. Figure 4-C: potential outliers identified from K-means clustering, DBSCAN, and Gaussian mixture models in studies reporting G11778A mutation rate among 3 primary LHON mutations. S. Figure 4-D: the Baujat plot of the influence of remaining studies reporting G11778A mutation rate among 3 primary LHON mutations after excluding potential outliers identified previously by K-means clustering, DBSCAN, and Gaussian mixture models. [file 1107866.f7.zip › Supplementary 4-C_SuppInfo.pdf]

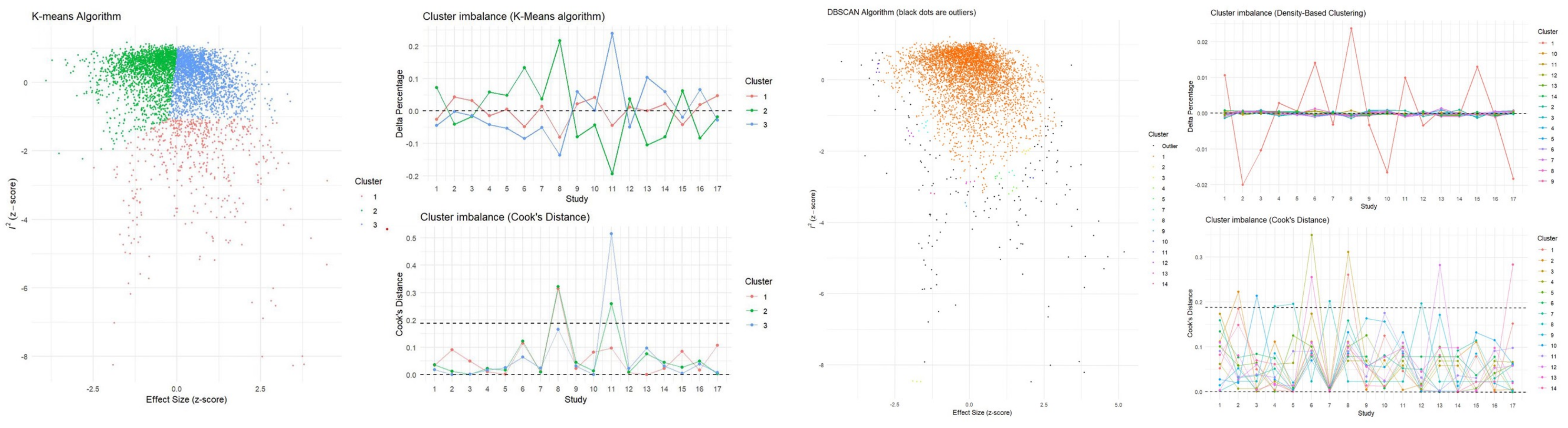

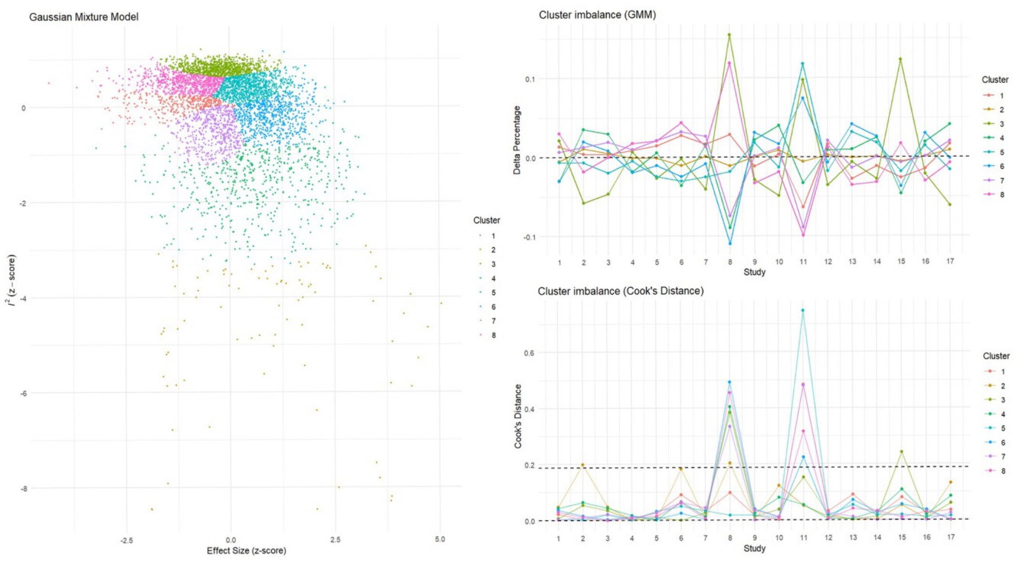

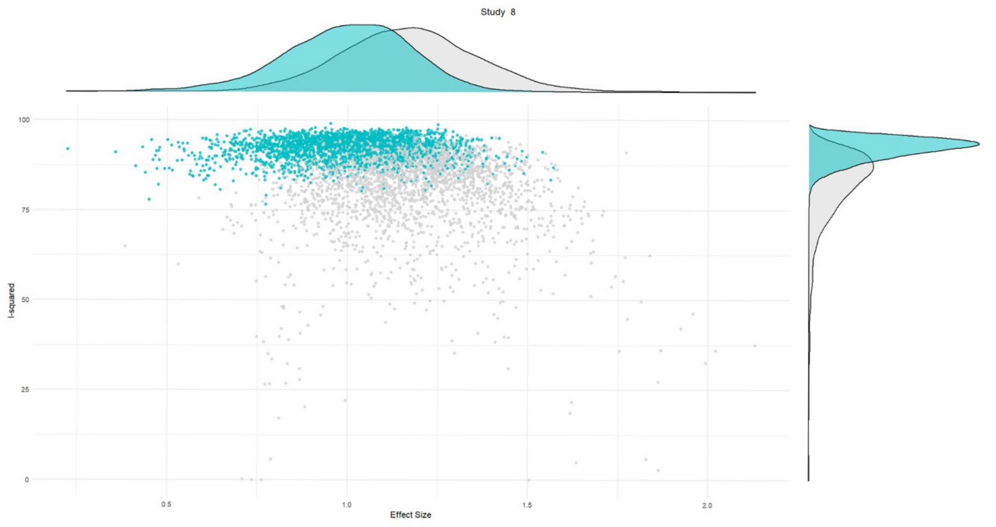

Spruijt et al. 2006

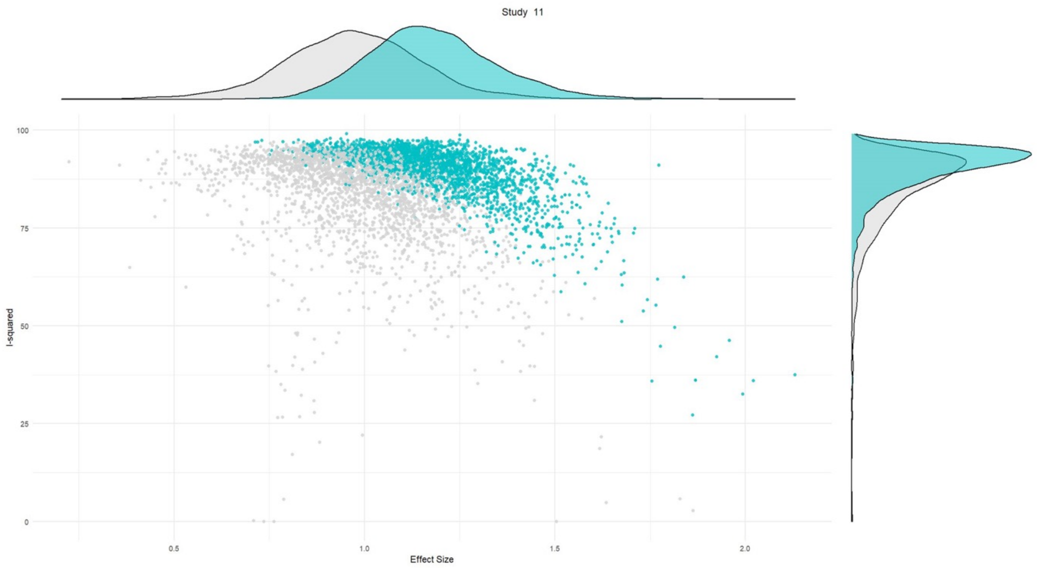

Ishikawa et al. 2021

Supplementary 4-C. Potential outliers identified from K-means clustering, DBSCAN, and Gaussian mixture models in studies reporting G11778A mutation rate among 3 primary LHON mutations.
