# Supplementary figures and images for: Leber's Hereditary Optic Neuropathy with Mitochondrial DNA Mutation G11778A: A Systematic Literature Review and Meta-Analysis
Source: Biomed Res Int. 2023 Jan 24;2023:1107866. doi: 10.1155/2023/1107866 (PMC9893526; doi:10.1155/2023/1107866)

Sorted by Proportion

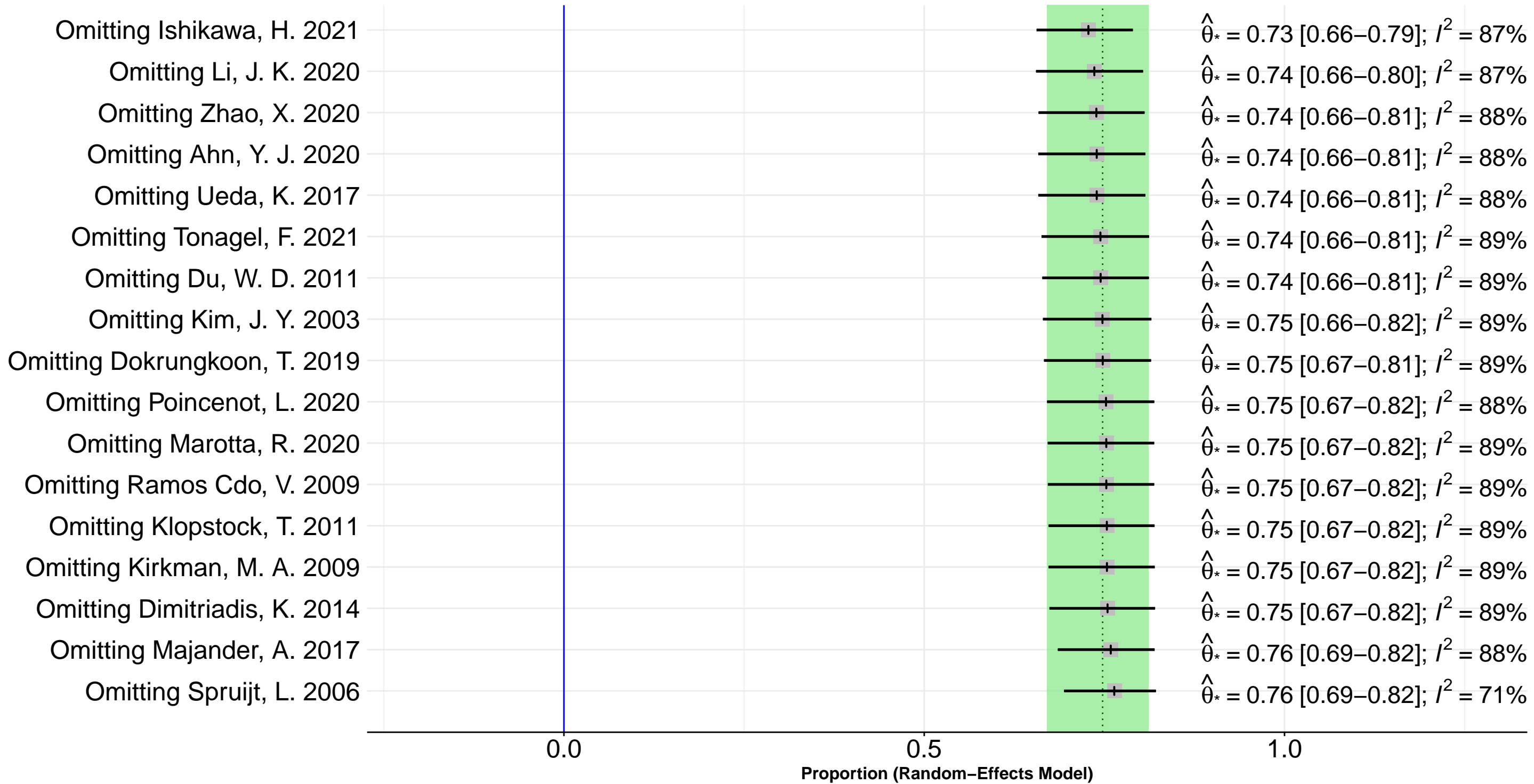

Supplement: Supplementary 7 — S. Figure 4-A: forest plot of G11778A mutation rate among 3 primary LHON mutations. S. Figure 4-B: leave-one-out analysis of studies reporting G11778A mutation rate among 3 primary LHON mutations. S. Figure 4-C: potential outliers identified from K-means clustering, DBSCAN, and Gaussian mixture models in studies reporting G11778A mutation rate among 3 primary LHON mutations. S. Figure 4-D: the Baujat plot of the influence of remaining studies reporting G11778A mutation rate among 3 primary LHON mutations after excluding potential outliers identified previously by K-means clustering, DBSCAN, and Gaussian mixture models. [file 1107866.f7.zip › S. Figure 4-B_SuppInfo.pdf]

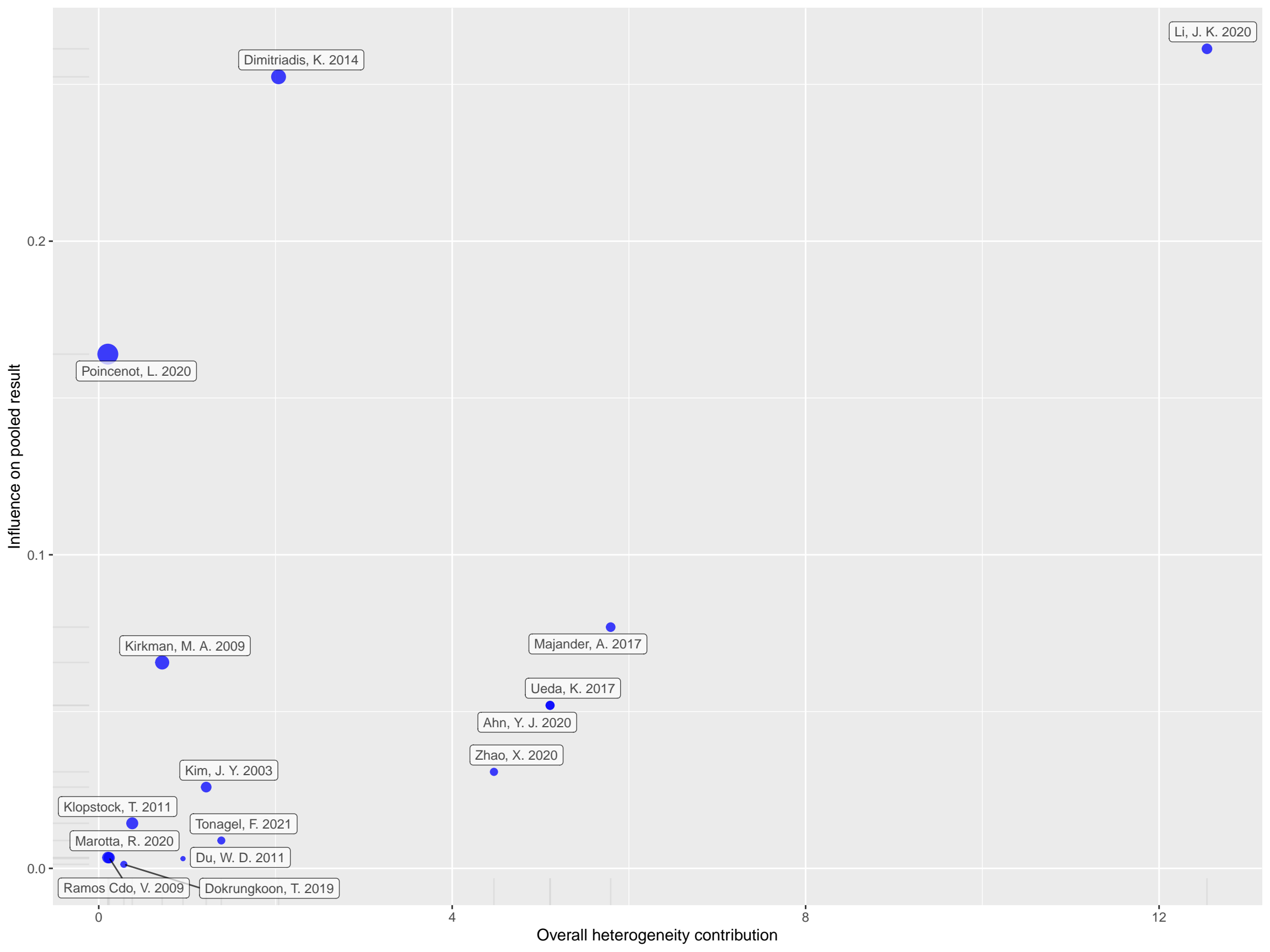

Supplement: Supplementary 7 — S. Figure 4-A: forest plot of G11778A mutation rate among 3 primary LHON mutations. S. Figure 4-B: leave-one-out analysis of studies reporting G11778A mutation rate among 3 primary LHON mutations. S. Figure 4-C: potential outliers identified from K-means clustering, DBSCAN, and Gaussian mixture models in studies reporting G11778A mutation rate among 3 primary LHON mutations. S. Figure 4-D: the Baujat plot of the influence of remaining studies reporting G11778A mutation rate among 3 primary LHON mutations after excluding potential outliers identified previously by K-means clustering, DBSCAN, and Gaussian mixture models. [file 1107866.f7.zip › S. Figure 4-D_SuppInfo.pdf]

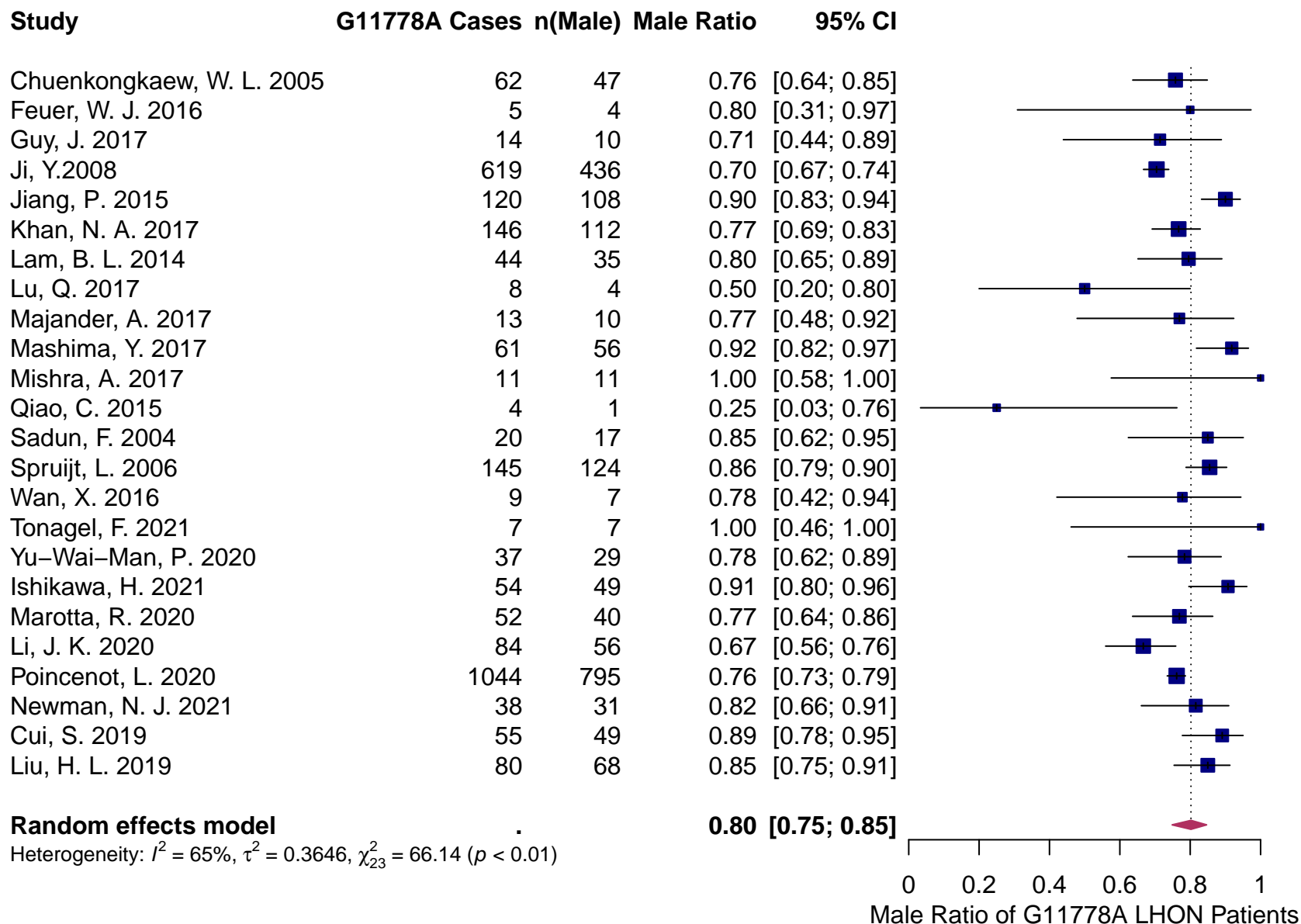

Supplement: Supplementary 8 — S. Figure 5-A: forest plot of male ratio of G11778A LHON patients. S. Figure 5-B: leave-one-out analysis of studies reporting male ratio of G11778A LHON patients. S. Figure 5-C: potential outliers identified from K-means clustering, DBSCAN, and Gaussian mixture models in studies reporting male ratio of G11778A LHON patients. [file 1107866.f8.zip › S. Figure 5-A_SuppInfo.pdf]

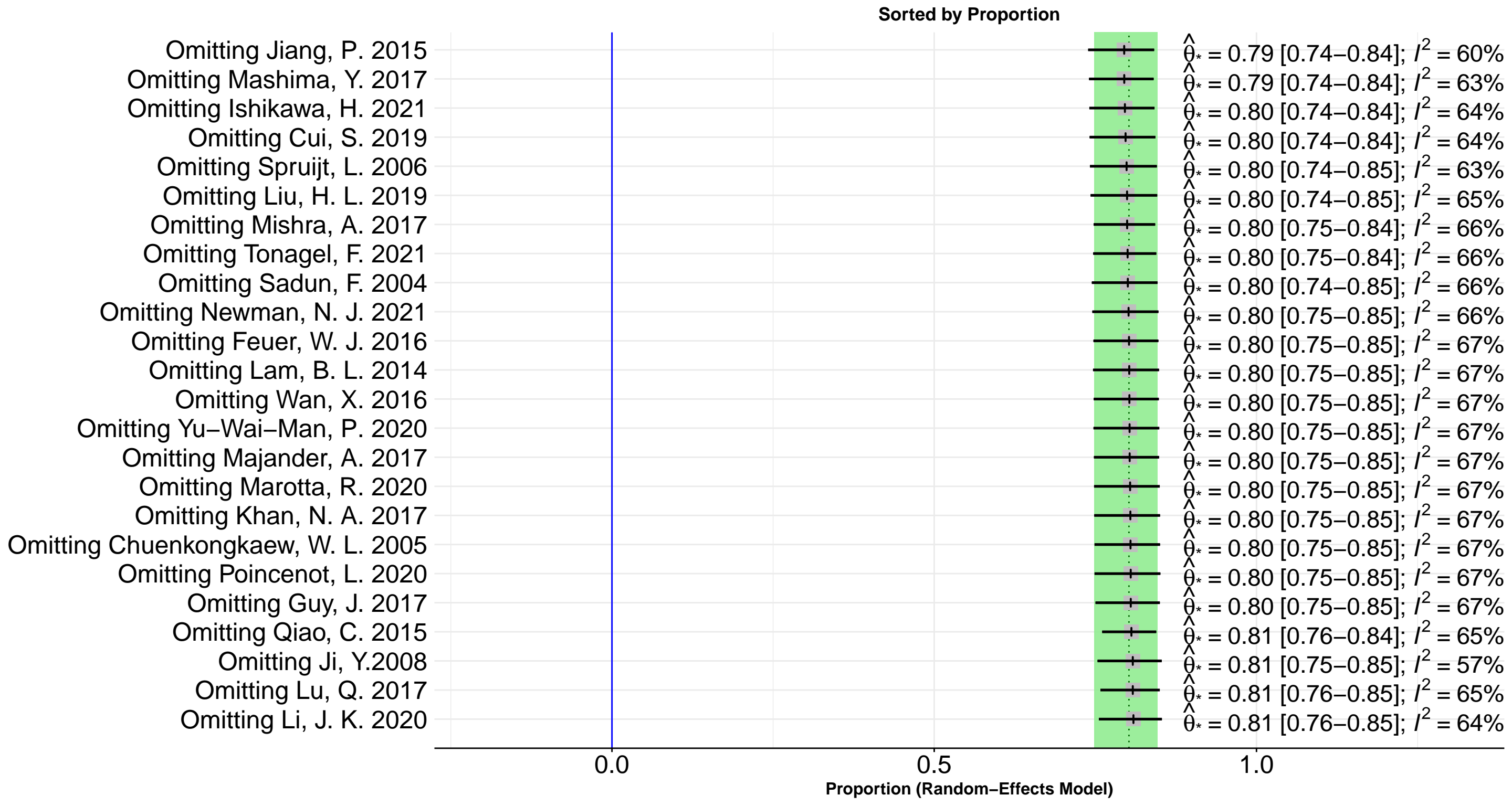

Supplement: Supplementary 8 — S. Figure 5-A: forest plot of male ratio of G11778A LHON patients. S. Figure 5-B: leave-one-out analysis of studies reporting male ratio of G11778A LHON patients. S. Figure 5-C: potential outliers identified from K-means clustering, DBSCAN, and Gaussian mixture models in studies reporting male ratio of G11778A LHON patients. [file 1107866.f8.zip › S. Figure 5-B_SuppInfo.pdf]

Sorted by Effect Size

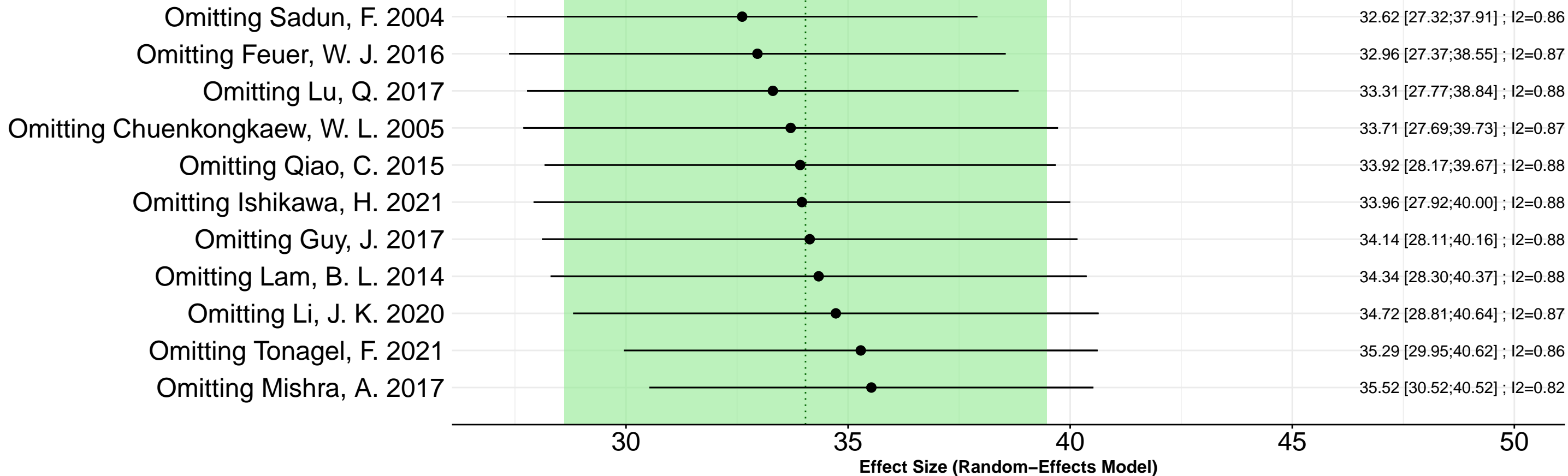

Supplement: Supplementary 9 — S. Figure 6-A: forest plot of age of G11778A LHON patients. S. Figure 6-B: leave-one-out analysis of studies reporting age of G11778A LHON patients. S. Figure 6-C: potential outliers identified from K-means clustering, DBSCAN, and Gaussian mixture models in studies reporting age of G11778A LHON patients. S. Figure 6-D: the Baujat plot of the influence of remaining studies reporting age of G11778A LHON patients after excluding potential outliers identified previously by K-means clustering, DBSCAN, and Gaussian mixture models. [file 1107866.f9.zip › S. Figure 6-B_SuppInfo.pdf]

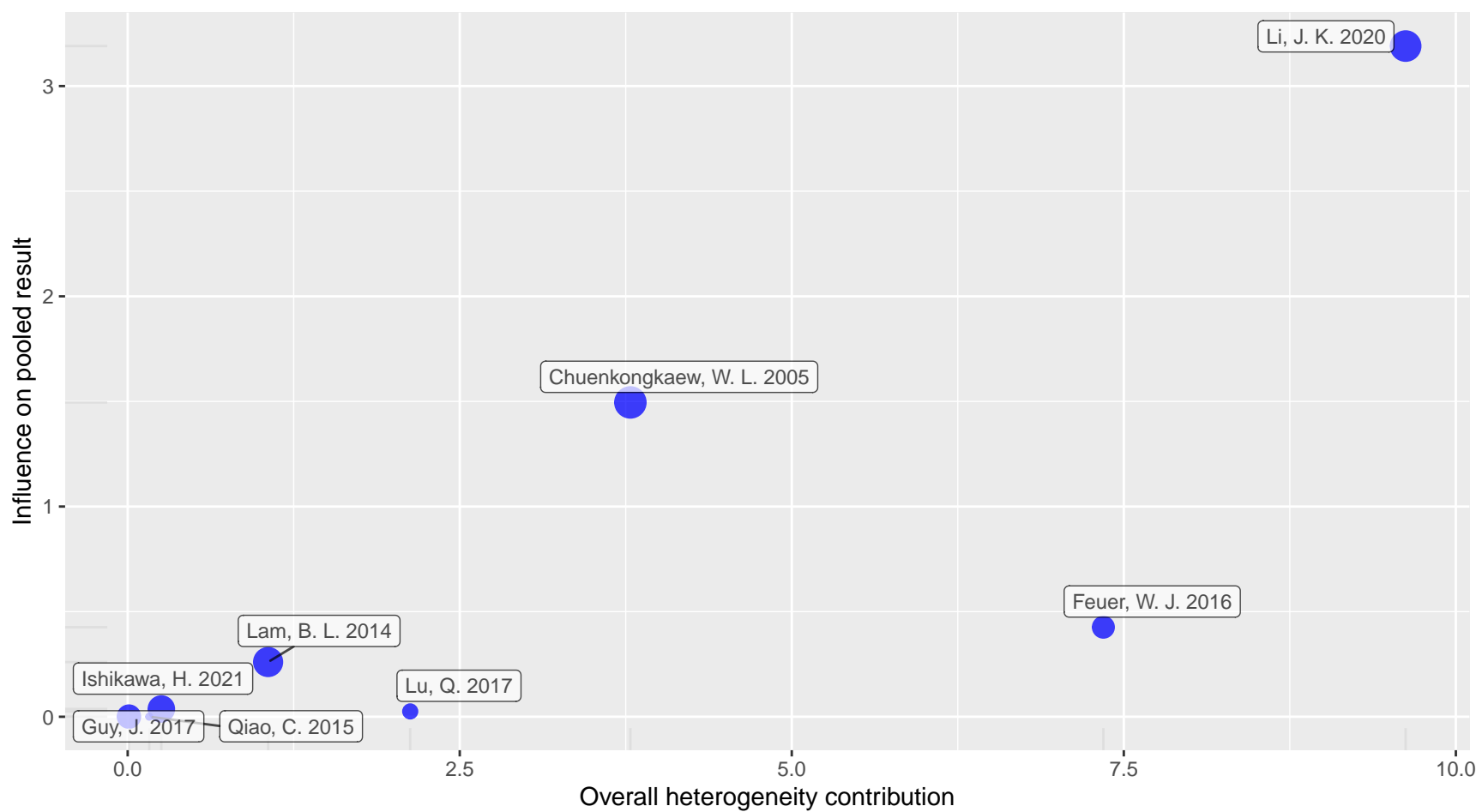

Supplement: Supplementary 9 — S. Figure 6-A: forest plot of age of G11778A LHON patients. S. Figure 6-B: leave-one-out analysis of studies reporting age of G11778A LHON patients. S. Figure 6-C: potential outliers identified from K-means clustering, DBSCAN, and Gaussian mixture models in studies reporting age of G11778A LHON patients. S. Figure 6-D: the Baujat plot of the influence of remaining studies reporting age of G11778A LHON patients after excluding potential outliers identified previously by K-means clustering, DBSCAN, and Gaussian mixture models. [file 1107866.f9.zip › S. Figure 6-D_SuppInfo.pdf]

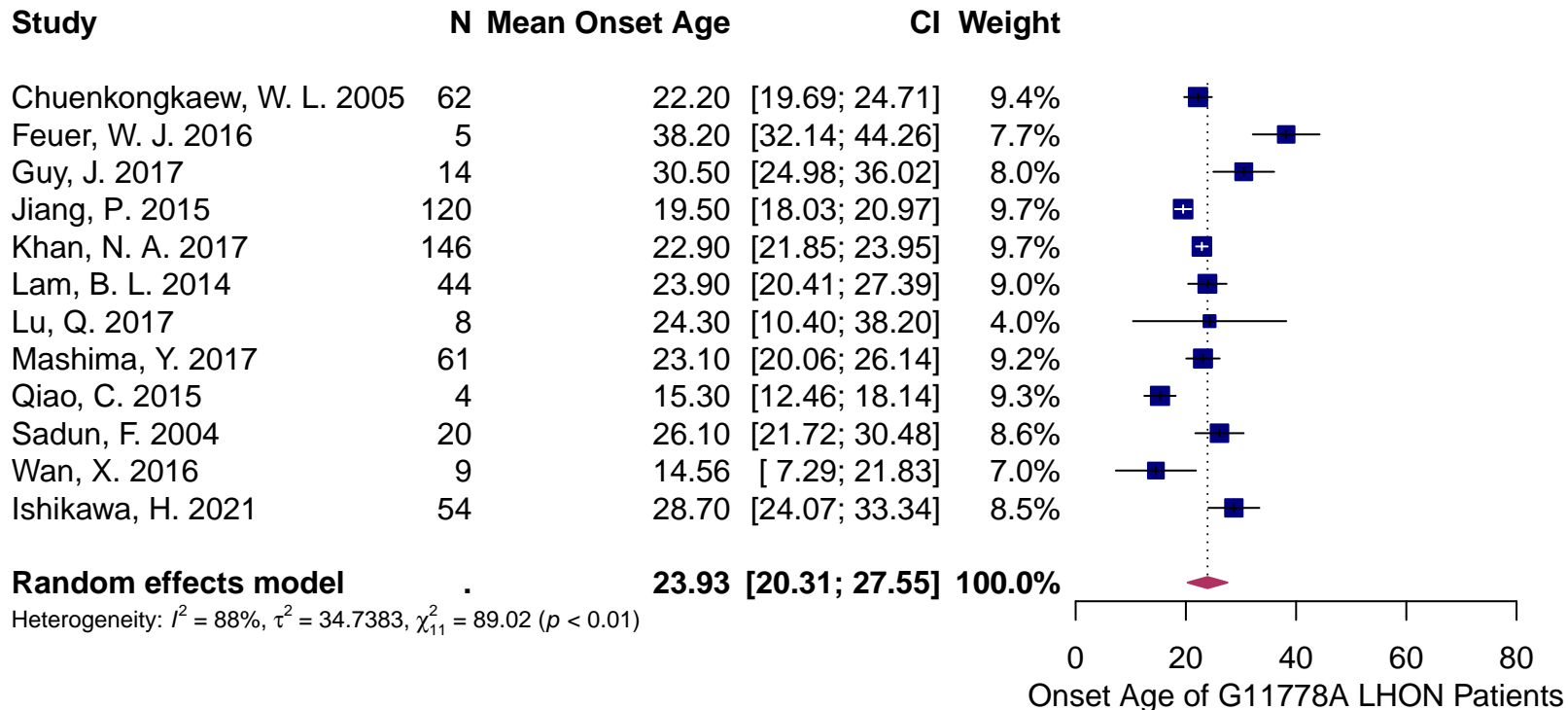

Supplement: Supplementary 10 — S. Figure 7-A: forest plot of onset age of G11778A LHON patients. S. Figure 7-B: leave-one-out analysis of studies reporting onset age of G11778A LHON patients. S. Figure 7-C: potential outliers identified from K-means clustering, DBSCAN, and Gaussian mixture models in studies reporting onset age of G11778A LHON patients. S. Figure 7-D: the Baujat plot of the influence of remaining studies reporting onset age of G11778A LHON patients after excluding potential outliers identified previously by K-means clustering, DBSCAN, and Gaussian mixture models. [file 1107866.f10.zip › S. Figure 7-A_SuppInfo.pdf]

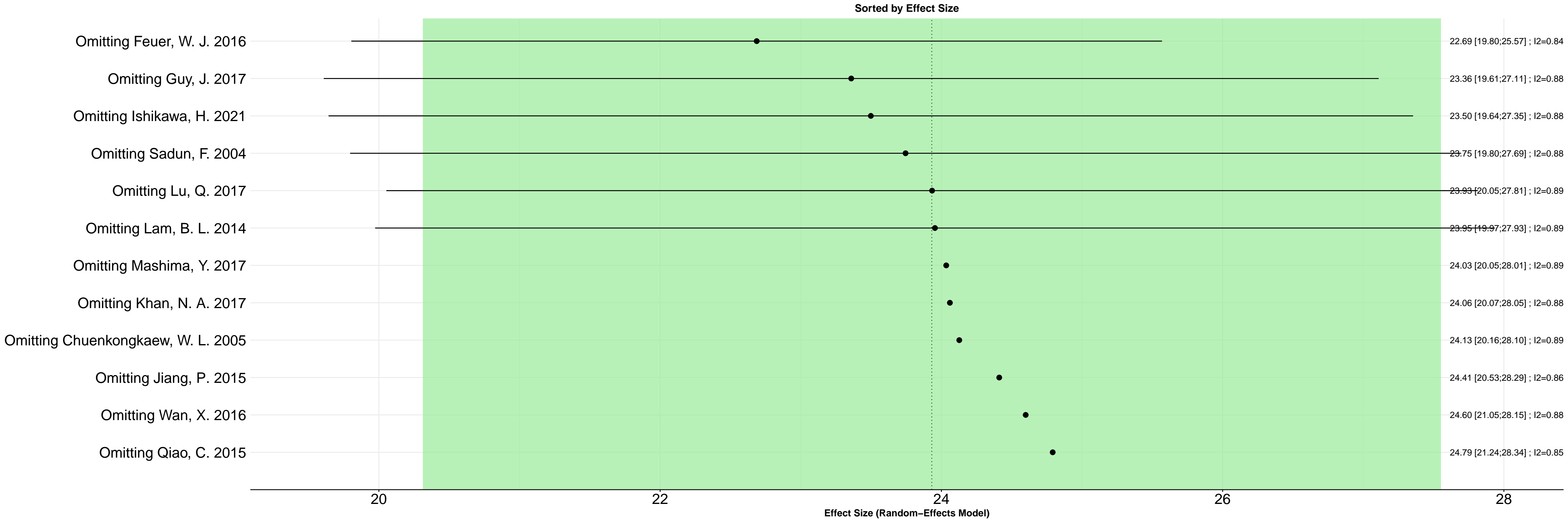

Supplement: Supplementary 10 — S. Figure 7-A: forest plot of onset age of G11778A LHON patients. S. Figure 7-B: leave-one-out analysis of studies reporting onset age of G11778A LHON patients. S. Figure 7-C: potential outliers identified from K-means clustering, DBSCAN, and Gaussian mixture models in studies reporting onset age of G11778A LHON patients. S. Figure 7-D: the Baujat plot of the influence of remaining studies reporting onset age of G11778A LHON patients after excluding potential outliers identified previously by K-means clustering, DBSCAN, and Gaussian mixture models. [file 1107866.f10.zip › S. Figure 7-B_SuppInfo.pdf]

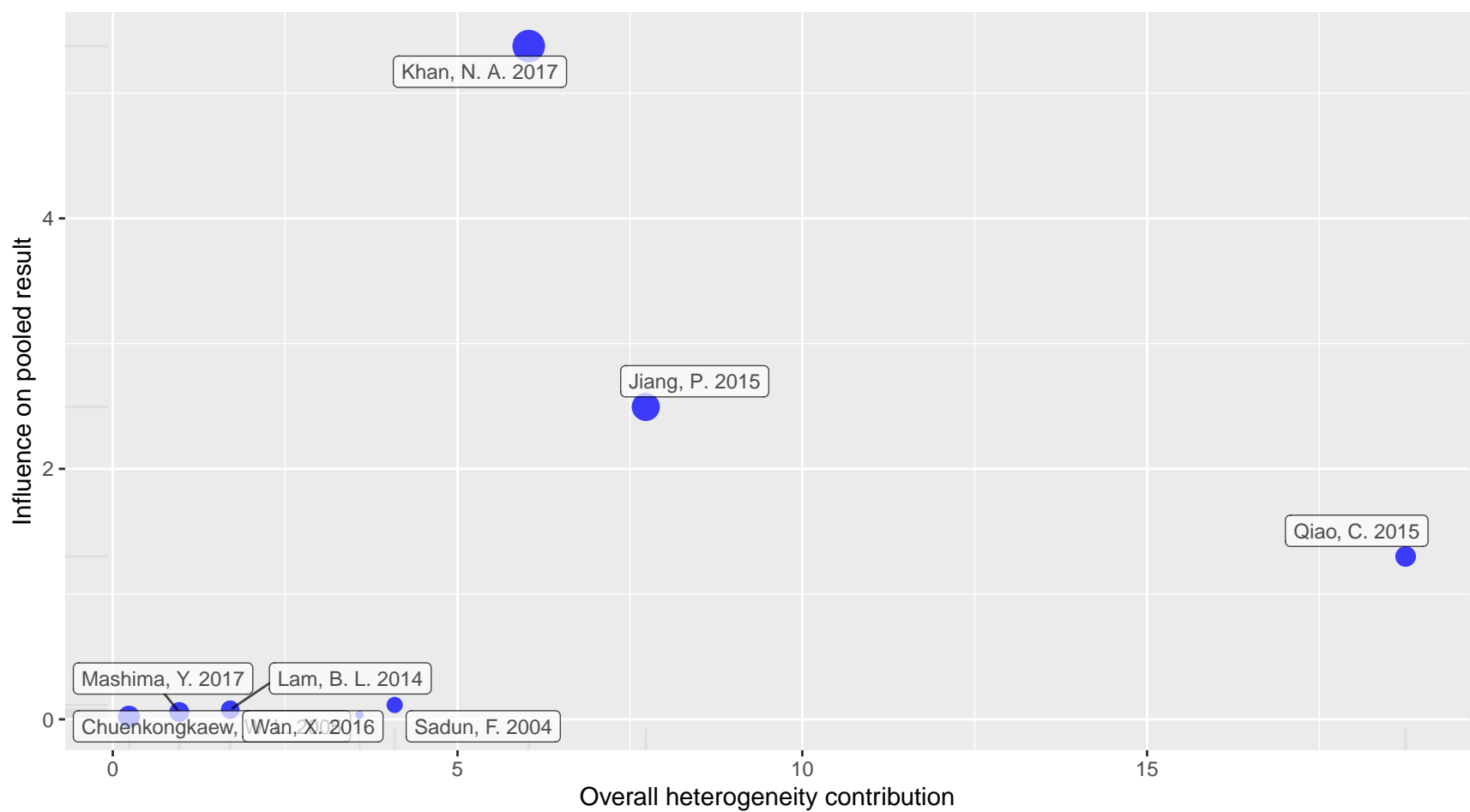

Supplement: Supplementary 10 — S. Figure 7-A: forest plot of onset age of G11778A LHON patients. S. Figure 7-B: leave-one-out analysis of studies reporting onset age of G11778A LHON patients. S. Figure 7-C: potential outliers identified from K-means clustering, DBSCAN, and Gaussian mixture models in studies reporting onset age of G11778A LHON patients. S. Figure 7-D: the Baujat plot of the influence of remaining studies reporting onset age of G11778A LHON patients after excluding potential outliers identified previously by K-means clustering, DBSCAN, and Gaussian mixture models. [file 1107866.f10.zip › S. Figure 7-D_SuppInfo.pdf]

Sorted by Effect Size

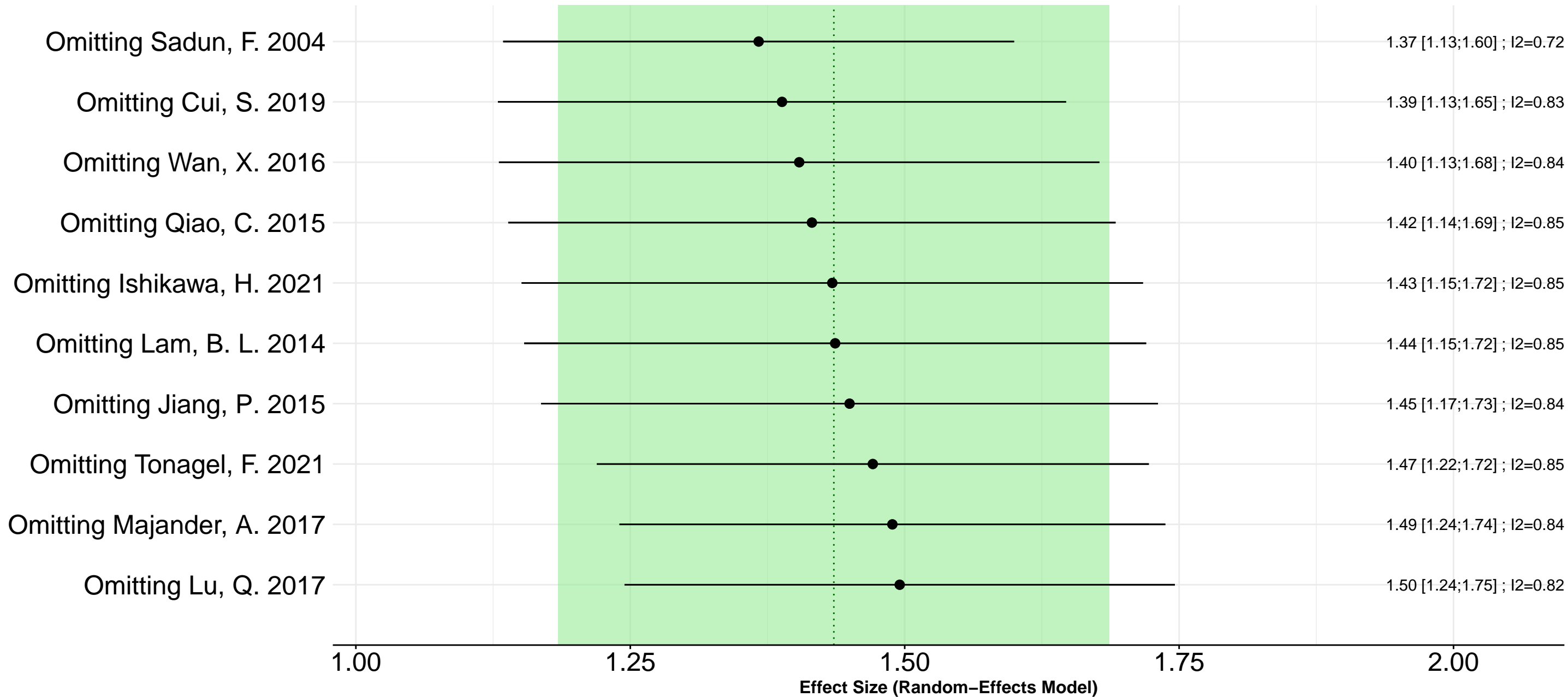

Supplement: Supplementary 11 — S. Figure 8-A: forest plot of visual acuity of G11778A LHON patients. S. Figure 8-B: leave-one-out analysis of studies reporting visual acuity of G11778A LHON patients. S. Figure 8-C: potential outliers identified from K-means clustering, DBSCAN, and Gaussian mixture models in studies reporting visual acuity of G11778A LHON patients. S. Figure 8-D: the Baujat plot of the influence of remaining studies reporting visual acuity of G11778A LHON patients after excluding potential outliers identified previously by K-means clustering, DBSCAN, and Gaussian mixture models. [file 1107866.f11.zip › S. Figure 8-B_SuppInfo.pdf]

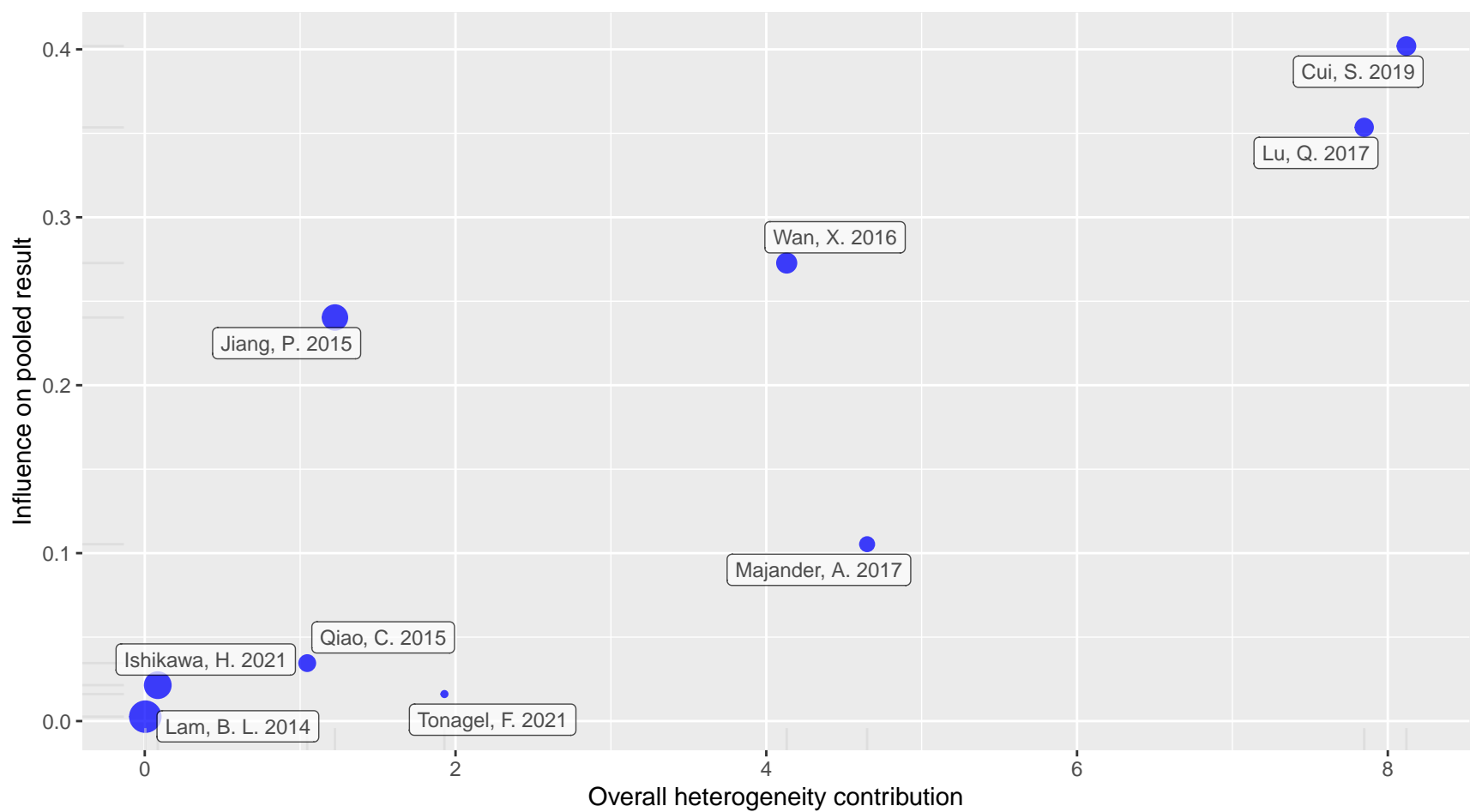

Supplement: Supplementary 11 — S. Figure 8-A: forest plot of visual acuity of G11778A LHON patients. S. Figure 8-B: leave-one-out analysis of studies reporting visual acuity of G11778A LHON patients. S. Figure 8-C: potential outliers identified from K-means clustering, DBSCAN, and Gaussian mixture models in studies reporting visual acuity of G11778A LHON patients. S. Figure 8-D: the Baujat plot of the influence of remaining studies reporting visual acuity of G11778A LHON patients after excluding potential outliers identified previously by K-means clustering, DBSCAN, and Gaussian mixture models. [file 1107866.f11.zip › S. Figure 8-D_SuppInfo.pdf]

# Sorted by Proportion

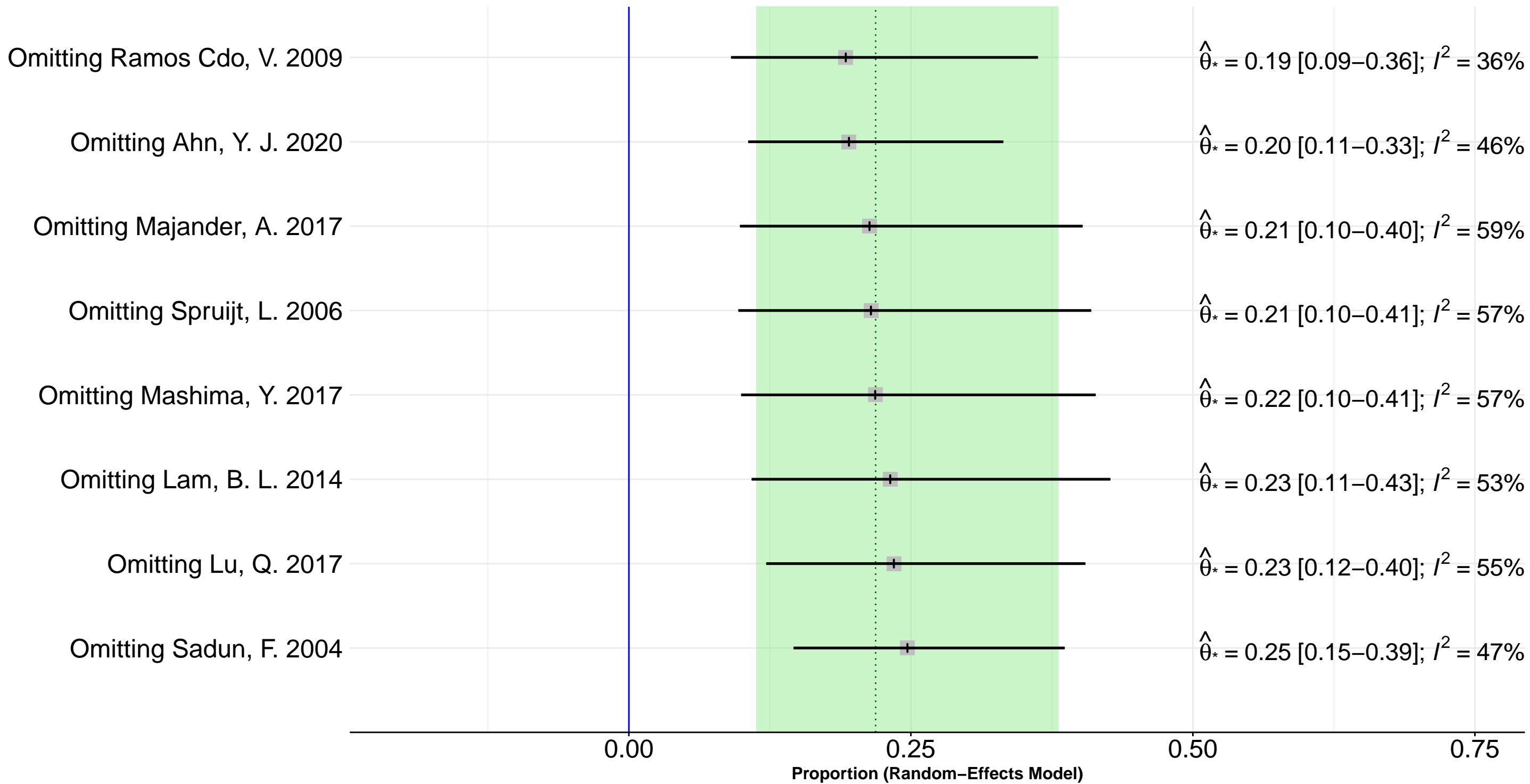

Supplement: Supplementary 12 — S. Figure 9-A: forest plot of visual recovery rate of G11778A LHON patients. S. Figure 9-B: leave-one-out analysis of studies reporting visual recovery rate of G11778A LHON patients. S. Figure 9-C: potential outliers identified from K-means clustering, DBSCAN, and Gaussian mixture models in studies reporting visual recovery rate of G11778A LHON patients. S. Figure 9-D: the Baujat plot of the influence of remaining studies reporting visual recovery rate of G11778A LHON patients after excluding potential outliers identified previously by K-means clustering, DBSCAN, and Gaussian mixture models. [file 1107866.f12.zip › S. Figure 9-B_SuppInfo.pdf]

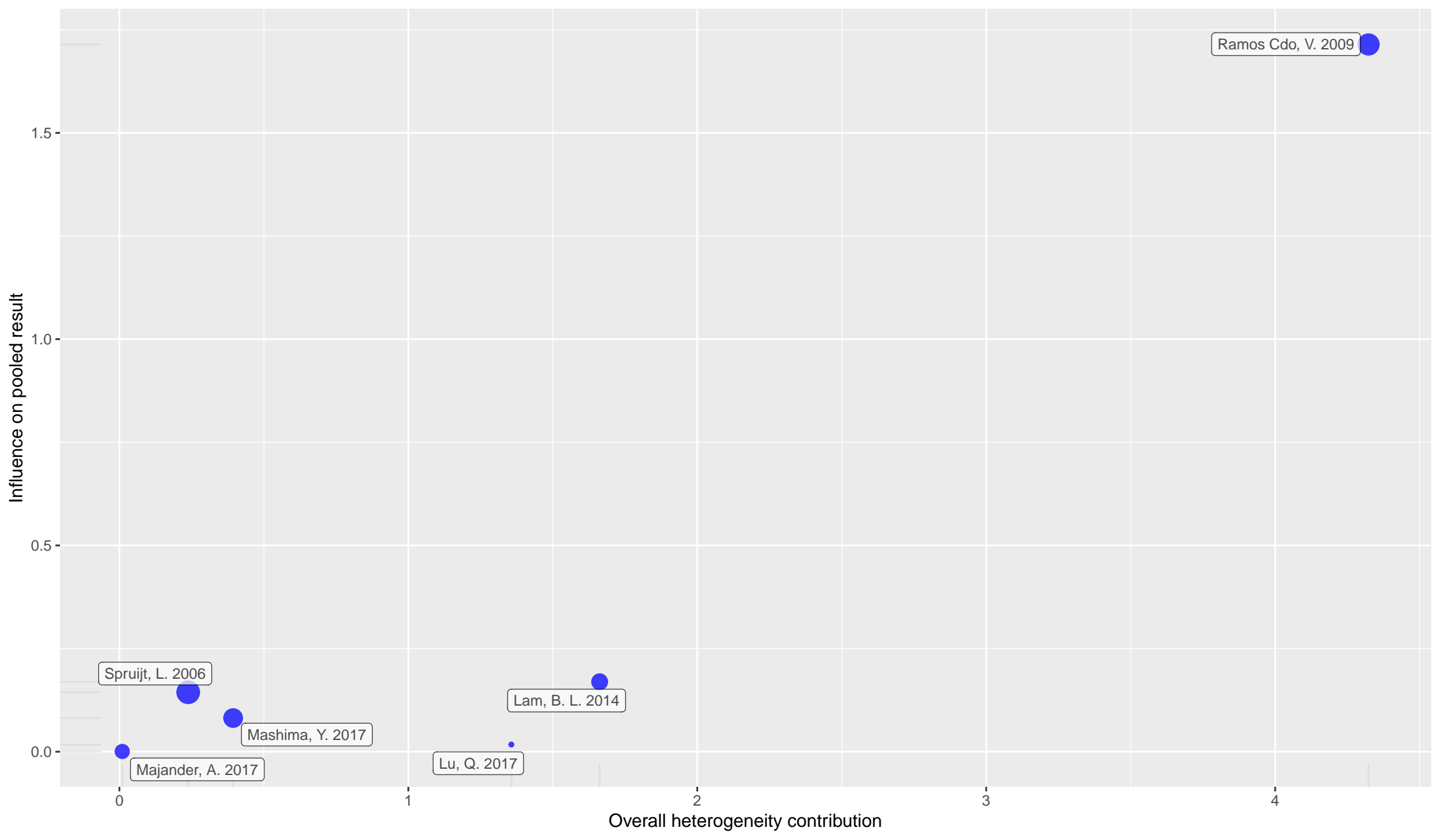

Supplement: Supplementary 12 — S. Figure 9-A: forest plot of visual recovery rate of G11778A LHON patients. S. Figure 9-B: leave-one-out analysis of studies reporting visual recovery rate of G11778A LHON patients. S. Figure 9-C: potential outliers identified from K-means clustering, DBSCAN, and Gaussian mixture models in studies reporting visual recovery rate of G11778A LHON patients. S. Figure 9-D: the Baujat plot of the influence of remaining studies reporting visual recovery rate of G11778A LHON patients after excluding potential outliers identified previously by K-means clustering, DBSCAN, and Gaussian mixture models. [file 1107866.f12.zip › S. Figure 9-D_SuppInfo.pdf]
