# Supplementary material for: Leber's Hereditary Optic Neuropathy with Mitochondrial DNA Mutation G11778A: A Systematic Literature Review and Meta-Analysis
Source: Biomed Res Int. 2023 Jan 24;2023:1107866. doi: 10.1155/2023/1107866 (PMC9893526; doi:10.1155/2023/1107866)
Supplement: Supplementary 8 — S. Figure 5-A: forest plot of male ratio of G11778A LHON patients. S. Figure 5-B: leave-one-out analysis of studies reporting male ratio of G11778A LHON patients. S. Figure 5-C: potential outliers identified from K-means clustering, DBSCAN, and Gaussian mixture models in studies reporting male ratio of G11778A LHON patients. [file 1107866.f8.zip › Supplementary 5-C_Supplnfo.pdf]

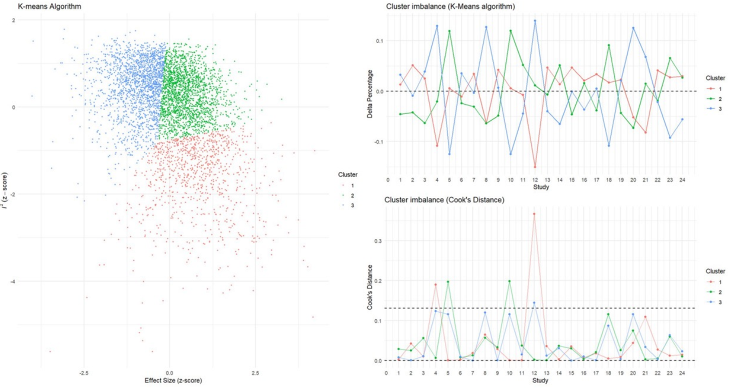

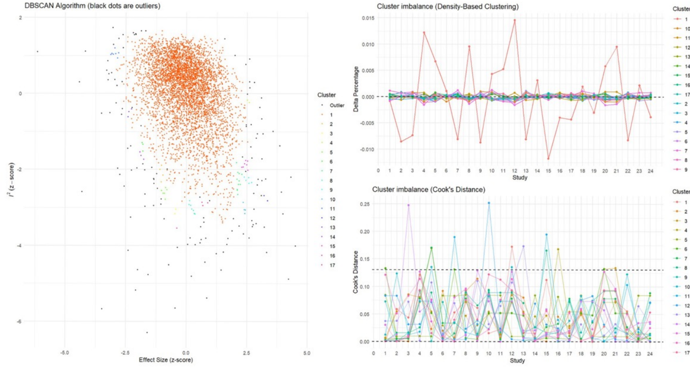

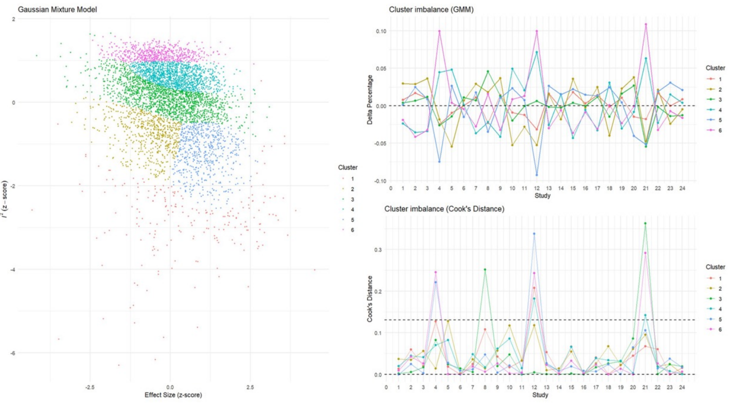

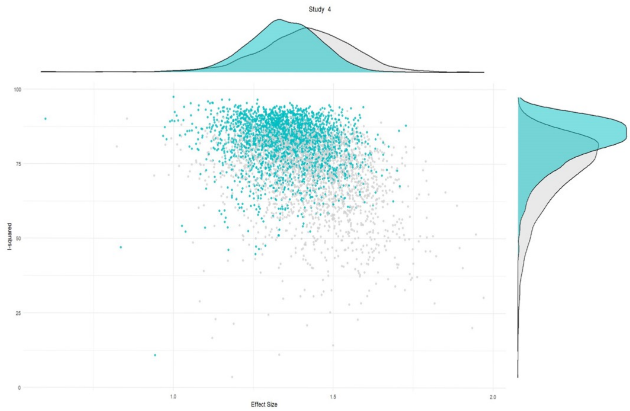

Ji et al. 2008

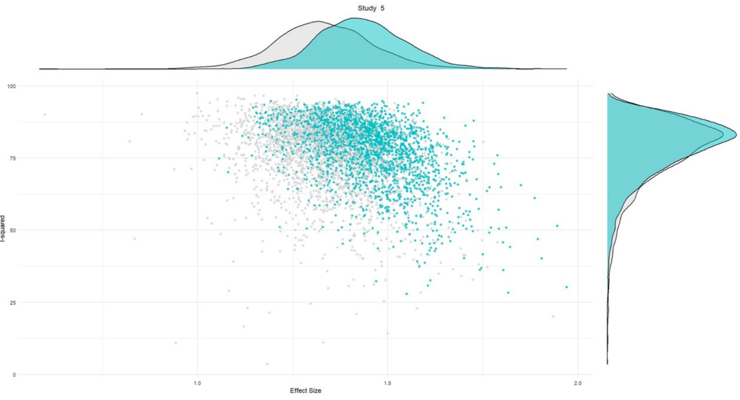

Jiang et al. 2015

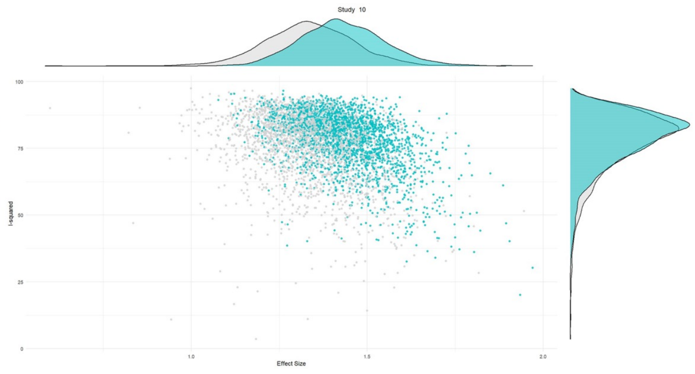

Mashima et al. 2017

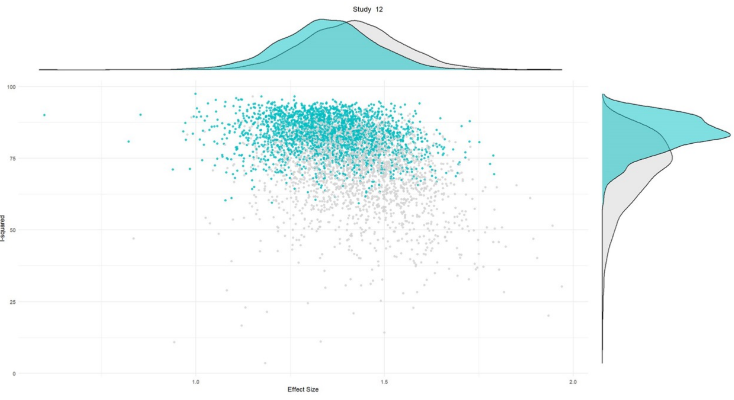

Qiao et al. 2015

Supplementary 5-C. Potential outliers identified from K-means clustering, DBSCAN, and Gaussian mixture models in studies reporting male ratio of G11778A LHON patients.
