# Supplementary material for: Leber's Hereditary Optic Neuropathy with Mitochondrial DNA Mutation G11778A: A Systematic Literature Review and Meta-Analysis
Source: Biomed Res Int. 2023 Jan 24;2023:1107866. doi: 10.1155/2023/1107866 (PMC9893526; doi:10.1155/2023/1107866)

Study	N	Mean Age	CI	Weight
Chuenkongkaew, W. L. 2005	62	37.30	[34.09; 40.51]	10.9%
Feuer, W. J. 2016	5	44.20	[36.91; 51.49]	9.4%
Guy, J. 2017	14	33.80	[27.47; 40.13]	9.8%
Lam, B. L. 2014	44	32.10	[28.26; 35.94]	10.7%
Lu, Q. 2017	8	45.80	[30.10; 61.50]	5.8%
Mishra, A. 2017	11	19.60	[14.40; 24.80]	10.3%
Qiao, C. 2015	4	40.30	[10.21; 70.39]	2.5%
Sadun, F. 2004	20	47.00	[39.55; 54.45]	9.3%
Tonagel, F. 2021	7	21.86	[15.73; 27.99]	9.9%
Ishikawa, H. 2021	54	35.34	[30.56; 40.12]	10.4%
Li, J. K. 2020	84	28.70	[25.28; 32.12]	10.8%
**Random effects model**	**.**	**34.04**	**[28.61; 39.48]**	**100.0%**

Heterogeneity: $I^2 = 87\%$, $\tau^2 = 67.8448$, $\chi^2_{10} = 77.54$ ($p < 0.01$)

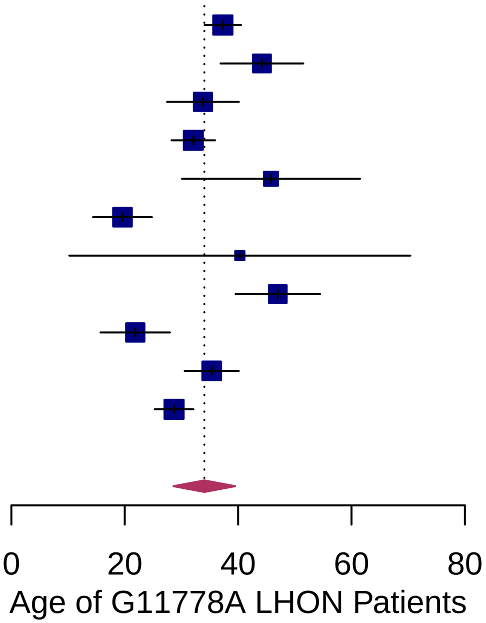

Supplement: Supplementary 9 — S. Figure 6-A: forest plot of age of G11778A LHON patients. S. Figure 6-B: leave-one-out analysis of studies reporting age of G11778A LHON patients. S. Figure 6-C: potential outliers identified from K-means clustering, DBSCAN, and Gaussian mixture models in studies reporting age of G11778A LHON patients. S. Figure 6-D: the Baujat plot of the influence of remaining studies reporting age of G11778A LHON patients after excluding potential outliers identified previously by K-means clustering, DBSCAN, and Gaussian mixture models. [file 1107866.f9.zip › S. Figure 6-A_SuppInfo.pdf]
