# Supplementary material for: Leber's Hereditary Optic Neuropathy with Mitochondrial DNA Mutation G11778A: A Systematic Literature Review and Meta-Analysis
Source: Biomed Res Int. 2023 Jan 24;2023:1107866. doi: 10.1155/2023/1107866 (PMC9893526; doi:10.1155/2023/1107866)
Supplement: Supplementary 9 — S. Figure 6-A: forest plot of age of G11778A LHON patients. S. Figure 6-B: leave-one-out analysis of studies reporting age of G11778A LHON patients. S. Figure 6-C: potential outliers identified from K-means clustering, DBSCAN, and Gaussian mixture models in studies reporting age of G11778A LHON patients. S. Figure 6-D: the Baujat plot of the influence of remaining studies reporting age of G11778A LHON patients after excluding potential outliers identified previously by K-means clustering, DBSCAN, and Gaussian mixture models. [file 1107866.f9.zip › Supplementary 6-C_SuppInfo.pdf]

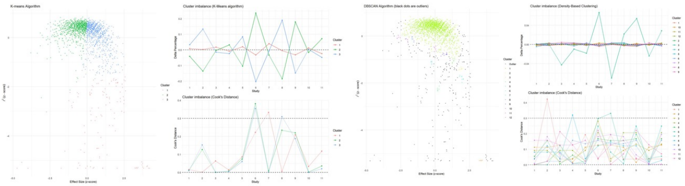

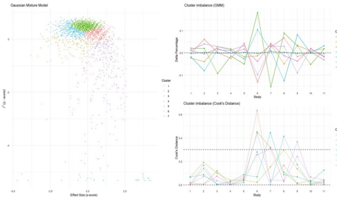

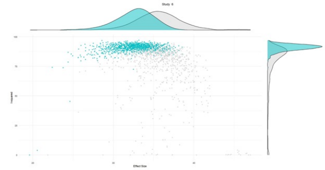

Mishra et al. 2017

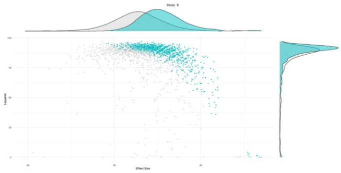

Sadun et al. 2004

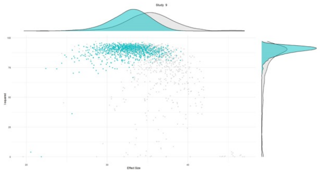

Tonagel et al. 2021

Supplementary 6-C. Potential outliers identified from K-means clustering, DBSCAN, and Gaussian mixture models in studies reporting age of G11778A LHON patients.
