# Supplementary material for: Leber's Hereditary Optic Neuropathy with Mitochondrial DNA Mutation G11778A: A Systematic Literature Review and Meta-Analysis
Source: Biomed Res Int. 2023 Jan 24;2023:1107866. doi: 10.1155/2023/1107866 (PMC9893526; doi:10.1155/2023/1107866)

| Study | Sample Size | Visual Acuity (LogMAR) | CI | Weight |
|---|---|---|---|---|
| Jiang, P. 2015 | 120 | 1.30 | [1.13; 1.47] | 11.5% |
| Lam, B. L. 2014 | 44 | 1.40 | [1.29; 1.51] | 11.9% |
| Lu, Q. 2017 | 7 | 0.92 | [0.58; 1.25] | 10.0% |
| Majander, A. 2017 | 13 | 0.88 | [0.41; 1.35] | 8.5% |
| Qiao, C. 2015 | 4 | 1.60 | [1.21; 1.99] | 9.4% |
| Sadun, F. 2004 | 20 | 2.05 | [1.80; 2.29] | 10.9% |
| Wan, X. 2016 | 9 | 1.69 | [1.41; 1.97] | 10.5% |
| Tonagel, F. 2021 | 7 | 0.85 | [0.09; 1.62] | 5.7% |
| Ishikawa, H. 2021 | 54 | 1.42 | [1.27; 1.58] | 11.6% |
| Cui, S. 2019 | 55 | 1.87 | [1.54; 2.19] | 10.1% |
| **Random effects model** | **.** | **1.44** | **[1.18; 1.69]** | **100.0%** |

Heterogeneity: $I^2 = 84\%$, $\tau^2 = 0.1353$, $\chi^2_9 = 54.59$ ($p < 0.01$)

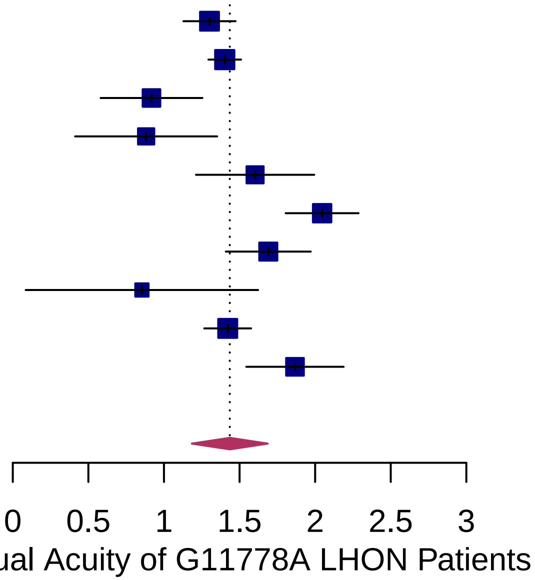

Supplement: Supplementary 11 — S. Figure 8-A: forest plot of visual acuity of G11778A LHON patients. S. Figure 8-B: leave-one-out analysis of studies reporting visual acuity of G11778A LHON patients. S. Figure 8-C: potential outliers identified from K-means clustering, DBSCAN, and Gaussian mixture models in studies reporting visual acuity of G11778A LHON patients. S. Figure 8-D: the Baujat plot of the influence of remaining studies reporting visual acuity of G11778A LHON patients after excluding potential outliers identified previously by K-means clustering, DBSCAN, and Gaussian mixture models. [file 1107866.f11.zip › S. Figure 8-A_SuppInfo.pdf]
