# Supplementary material for: Leber's Hereditary Optic Neuropathy with Mitochondrial DNA Mutation G11778A: A Systematic Literature Review and Meta-Analysis
Source: Biomed Res Int. 2023 Jan 24;2023:1107866. doi: 10.1155/2023/1107866 (PMC9893526; doi:10.1155/2023/1107866)

Study	Total	Recovered	Recovery Rate	95% CI
Lam, B. L. 2014	29	4	0.14	[0.05; 0.31]
Lu, Q. 2017	8	0	0.00	[0.00; 0.50]
Majander, A. 2017	13	3	0.23	[0.08; 0.52]
Mashima, Y. 2017	40	8	0.20	[0.10; 0.35]
Ramos Cdo, V. 2009	45	17	0.38	[0.25; 0.53]
Sadun, F. 2004	20	0	0.00	[0.00; 0.29]
Spruijt, L. 2006	82	18	0.22	[0.14; 0.32]
Ahn, Y. J. 2020	7	4	0.57	[0.23; 0.86]
**Random effects model**	**.**		**0.22**	**[0.11; 0.38]**

Heterogeneity: $I^2 = 52\%$, $\tau^2 = 0.8440$, $\chi^2_7 = 14.67$ ($p = 0.04$)

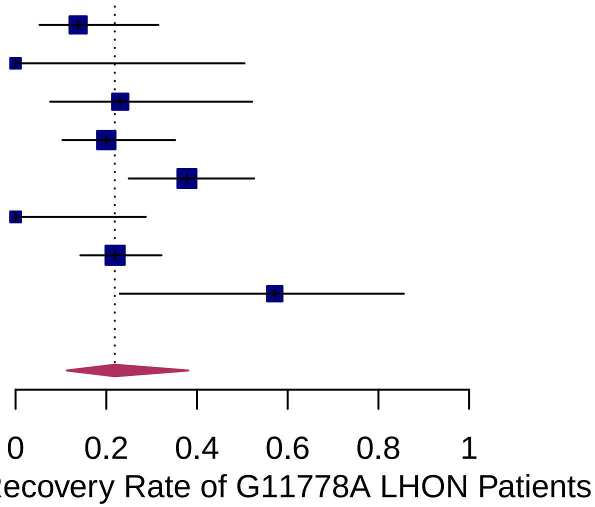

Supplement: Supplementary 12 — S. Figure 9-A: forest plot of visual recovery rate of G11778A LHON patients. S. Figure 9-B: leave-one-out analysis of studies reporting visual recovery rate of G11778A LHON patients. S. Figure 9-C: potential outliers identified from K-means clustering, DBSCAN, and Gaussian mixture models in studies reporting visual recovery rate of G11778A LHON patients. S. Figure 9-D: the Baujat plot of the influence of remaining studies reporting visual recovery rate of G11778A LHON patients after excluding potential outliers identified previously by K-means clustering, DBSCAN, and Gaussian mixture models. [file 1107866.f12.zip › S. Figure 9-A_SuppInfo.pdf]
